# Respiratory syncytial virus hospitalization costs, rates, and seasonality in Asia: a systematic review and meta-analysis

**DOI:** 10.1016/j.eclinm.2025.103350

**Published:** 2025-07-10

**Authors:** Luis J. Ponce, Thaddaeus Wu, Darren Junfeng Sim, Jo Yi Chow, Liang En Wee, Po Ying Chia, David Chien Boon Lye, Yee-Sin Leo, Jue Tao Lim

**Affiliations:** a Lee Kong Chian School of Medicine, Nanyang Technological University, Singapore; b National Centre for Infectious Diseases, Singapore; c Department of Infectious Diseases, Tan Tock Seng Hospital, Singapore; d Department of Medicine, Yong Loo Lin School of Medicine, National University of Singapore, Singapore; e Saw Swee Hock School of Public Health, National University of Singapore, Singapore; f Department of Infectious Diseases, Singapore General Hospital, Singapore

**Keywords:** Respiratory syncytial virus, Asia, RSV seasonality, Hospitalization costs, Public health burden, Low- and middle-income countries

## Abstract

**Background:**

RSV is a major cause of morbidity and a large public health threat, yet its burden in Asia remains poorly characterized, limiting effective public health planning. This study's objectives were to systematically review RSV hospitalization rates, seasonality, and medical costs in Asia.

**Methods:**

We conducted a systematic review and meta-analysis, searching five major public health databases (PubMed, Cochrane Library, Web of Science, Embase, Scopus) for peer-reviewed articles published from January 2005 to the last search date of 16 April 2025. Studies reporting RSV incidence, hospitalization rates, costs, or seasonality in Asia were included, regardless of healthcare setting or study design. Exclusions included studies without primary data, data from outside of Asia, and those lacking clear RSV case definitions. Two independent reviewers performed data extraction and quality assessment using JBI and TRIPOD critical appraisal tools. A meta-analysis and publication bias analysis of mean direct medical costs was performed. Data were synthesized by seasonality, costs, and hospitalization rates.

**Findings:**

Of 1231 screened studies, 159 met inclusion criteria. RSV seasonality varied: temperate regions showed winter peaks, while tropical regions had more variable patterns. RSV-associated hospitalization rates ranged widely, from 0.42 per 1000 person-years among the elderly in Thailand to 124 per 1000 children-years among infants <2 years in the Philippines. Overall, rates declined with age. RSV direct inpatient medical costs ranged from US$126–2448 in LMICs to US$838–3402 in high-income countries. 104 (65%) of studies were classified as having a low risk of bias, while 55 (35%) received a moderate risk of bias score.

**Interpretation:**

This study highlights the significant burden of RSV in Asia, particularly among young children, and highlights substantial variation in seasonality and economic impact across the region. The findings emphasize the need for region-specific RSV data to inform targeted prevention strategies and healthcare resource allocation. High heterogeneity in cost estimates suggests variability in healthcare access and economic conditions, warranting further investigation.

**Funding:**

Jue Tao Lim is supported by the Lee Kong Chian School of Medicine—Ministry of Education Start-Up Grant. YSL is funded by the National Centre for Infectious Diseases Department Research Fund.

**Research in context**

**Evidence before this study**

RSV is a well-established cause of severe respiratory infections, particularly in infants and older adults, but its burden remains poorly characterized in many regions, including Asia. While global estimates of RSV incidence and economic burden exist, most data come from high-income settings, with limited systematic analyses of region-specific trends in Asia. Previous studies have described RSV seasonality and incidence and RSV-associated costs in parts of the continent, usually country-specific or in a handful of Asian countries. However, no review has integrated burden estimates across multiple outcomes, including seasonality, incidence, and hospitalization costs, in the entire continent. Additionally, RSV-related healthcare costs have been underreported or inconsistently assessed, particularly in low- and middle-income countries, where surveillance systems and diagnostic capacities vary widely.

**Added value of this study**

This study provides the most comprehensive synthesis of RSV burden in Asia, integrating epidemiological and economic data across diverse healthcare settings. Unlike prior work, this review quantifies age-specific incidence rates, showing that infants under one year of age experience the highest burden, and highlights substantial regional variation in RSV seasonality between temperate and tropical climates. The findings also include a meta-analysis of hospitalization costs, addressing critical gaps in cost estimates by differentiating between high-income and lower-resource settings. Furthermore, by assessing risk of bias, this study identifies key limitations in existing research, such as selection bias in incidence studies and inconsistencies in cost reporting methods, which have implications for data interpretation and public health planning.

**Implications of all the available evidence**

The variability in RSV seasonality suggests that universal seasonal rollout vaccine strategies may not be effective due to the large variation between and within countries regarding RSV seasonality, and that maternal and pediatric RSV immunization programs should be tailored to local epidemiological patterns. The disproportionately high burden among infants highlights the importance of early-life protection strategies, including maternal immunization and monoclonal antibody prophylaxis. The economic burden of RSV-associated medical costs emphasizes the need for cost-effectiveness analyses to inform vaccine rollout and resource allocation, especially in lower-income settings. Future research should focus on standardizing RSV data collection across diverse healthcare systems to improve burden estimates and optimize prevention strategies.

## Introduction

Respiratory syncytial virus (RSV) is one of the leading causes of severe respiratory infections worldwide, particularly among newborns, infants, young children, the elderly, and immunocompromised individuals, resulting in millions of hospitalizations each year.^1^ It is a major contributor of lower respiratory tract infections (LRTIs) in infants and young children, often progressing to pneumonia or severe bronchiolitis.^2^ Globally, RSV is associated with significant morbidity and mortality, imposing a substantial burden on healthcare systems. In 2005, an estimated 33.8 million episodes of RSV-related acute lower respiratory tract infections (ALRIs) occurred in children under five years of age, resulting in up to 199,000 deaths.^3^ A decade later, in 2015, the numbers remained alarmingly similar, with 33.1 million episodes and 118,200 deaths, demonstrating the persistent public health challenge caused by RSV.^1^

RSV burden and seasonality vary widely across geographic regions due to climatic and environmental factors. In temperate regions, RSV epidemics typically occur during colder months and last 2–5 months, whereas in tropical areas, outbreaks often coincide with the rainy season and last longer.^4^ For example, RSV epidemics in Mozambique and Malaysia can last up to 10 months, compared to shorter seasons in Spain or the UK.^5^^,^^6^ Within Asia, countries such as Singapore and Malaysia show transmission patterns tied to rainfall and temperature, but these are less predictable than in temperate zones, complicating prevention strategies.^7^

Despite RSV's significant public health impact, its burden in the continent of Asia remains poorly understood. Routine testing is uncommon, leading to frequent underdiagnoses and misclassifications of RSV-related hospitalizations.^8^ Lack of region-specific data, especially in low- and middle-income countries (LMICs), leads to difficulty in designing effective interventions tailored to local epidemiological patterns. Since 2005, there has been a rise in literature regarding the public health burden of RSV. However, many of these studies focus on city- or country-level epidemiological characteristics of RSV. Few research teams have conducted larger investigations into the burden of RSV at a continental or global scale, and even these studies have not comprehensively captured a large proportion of Asia.^9^, ^10^, ^11^ This knowledge gap is particularly concerning given Asia's large and diverse population. Limited understanding of RSV seasonality and disease burden further impede efficient public health interventions and resource allocation.

Recent advances in RSV vaccine development have created an opportunity to address the global burden of this virus. Newly approved vaccines, including Pfizer's RSVpreF (ABRYSVO), GSK's Arexvy, and Moderna's mResvia, demonstrate high efficacy in preventing severe outcomes in older adults and maternal immunization strategies have been found to confer protective effects on newborns.^12^, ^13^, ^14^, ^15^ Despite progress, significant challenges persist in pediatric vaccine trials and equitable vaccine distribution in LMICs. Current estimates of RSV management costs in LMICs range from US$92 to US$4114 per severe episode, but this broad range reflects a lack of robust data, particularly in resource-poor settings.^16^ Modeling studies suggest that a comprehensive understanding of RSV seasonality, transmission patterns, and economic impact is critical for refining intervention cost-effectiveness and resource allocation.^17^, ^18^, ^19^, ^20^ For instance, maternal vaccines have been shown to prevent up to 57% of infant hospitalizations, with estimated savings of US$1.96–2.68 million in hospital-related costs in the Netherlands.^18^^,^^21^ Addressing these data gaps is vital to ensure accurate assessment on the cost-effectiveness of RSV prevention measures.

The timing of a systematic review and meta-analysis (SRMA) regarding the burden of RSV in Asia is hence critical due to such advancements in RSV vaccines and preventive strategies. As many Asian countries are evaluating the cost-effectiveness of RSV interventions amid rising healthcare expenses, better economic considerations must be assessed to drive such public health decision-making. Furthermore, the region's diverse climate conditions lead to varying seasonality patterns, underscoring the importance of region-specific data to optimize vaccine rollout and other preventive measures.

As such, this study seeks to provide a comprehensive analysis of the transmission patterns and burden of RSV in Asia, with a focus on seasonality, hospitalization rates and incidence, and medical costs. By addressing critical knowledge gaps, this systematic review aims to inform evidence-based immunization strategies and public health policies tailored to the continent's unique demographic and socioeconomic landscape. These findings have the potential to drive more effective interventions, reduce RSV-related morbidity and mortality, and alleviate the broader healthcare and economic burden across Asian nations.

## Methods

### Search strategy and selection criteria

A systematic review was conducted by three reviewers (LJP, DJS, TW) working independently to identify studies examining the burden of RSV among regions of Asia. Five major scientific databases were searched to capture as many relevant studies as possible: Embase, Cochrane Library, Scopus, Web of Science, and PubMed. The following terms were search for appearance anywhere in the text: “RSV” or “respiratory syncytial virus” and “Asia” or any Asian country and “incidence” or “cost” (and any variation of the word) or “seasonality.” The full search entries can be found in the Supplementary Materials. The search was designed to capture studies reporting on RSV incidence, hospitalization rates, hospitalization costs, and seasonality trends across diverse healthcare settings in Asia. Inclusion criteria involved studies with primary data on RSV infection incidence, hospitalization outcomes, or seasonality patterns, regardless of study design or healthcare context. Moreover, we only included studies published from 1 January 2005 (until 16 April 2025), as this year marked the beginning of a rise in RSV burden and publications. To ensure data reliability and relevance, only studies with a sample size (i.e., total number of cases in the study) of at least 50 RSV-infected individuals were included. Articles were excluded if RSV was reported solely as a co-infection, if they lacked specific data on seasonality, incidence/hospitalization rates, or economic burden, or if the data did not include any Asian countries. We did not have any language limitations, but expert opinions, protocols, and studies with redundant data (e.g., systematic reviews of which all relevant studies were already found in this present review) were also excluded to maintain consistency. Studies were also excluded if they had incomplete or unclear data, such as reports of seasonality that did not observe every month of the year, or data reporting outcomes of multiple pathogens together without specific data about RSV. During quality assessments, any study deemed as having a high risk of bias would have also been excluded. Data were collected for each report by at least two reviewers working independently. Any differences in data were discussed and settled with a third reviewer. No automation tools were used in this systematic review. All included studies are summarized in Table S1, including the study populations, study years, study designs relevant to our desired outcomes, measurements, and risks of bias.

### Data normalization and classification

Studies were classified based on reported population characteristics including age groups and country or region where the study was performed to standardize comparisons across hospitalization rates, costs, and seasonality. Months observing highest RSV transmission—hereafter referred to as “peaks”—were identified using reported incidence or hospital admission rates due to RSV infection, and studies were considered to report peak months of transmission if clear trends were evident in their datasets.

Hospitalization costs were standardized to a common currency (United States dollars, US$) and to the latest year available (2024) as of May 2025 for cross-study comparability using the World Bank's Consumer Price Index and exchange rates.^22^ Costs were further categorized as direct medical costs (e.g., hospitalization charges, ICU costs, medications, diagnostic tests) and indirect costs (e.g., lost productivity, caregiver costs, and transportation). Indirect costs were not included in this SRMA. Intensive care unit (ICU) and non-ICU hospitalization costs were analyzed together to capture the full variation in RSV-attributable medical costs. If studies did not explicitly report this classification, cost data were categorized based on the nature of the healthcare services described. All reported costs are for the general population and are not stratified by age or any other demographic.

Incidence and hospitalization rates were further categorized by specific disease outcomes, including RSV-ALRI (acute lower respiratory tract infection caused by RSV), RSV-ARTI (acute respiratory tract infection caused by RSV), and severe ALRI (severe acute lower respiratory tract infection requiring intensive care). To account for variations in the populations studied, rates were classified according to demographic groups, including infants (children under 6 months and under 1 year of age), children (up to 5 years of age), pregnant women, the general population (unspecified ages or encompassing all ages), and the elderly (aged 65 years and above). These classifications ensured consistency in comparing RSV burden across different populations, healthcare settings, and disease outcomes. Information on how hospitalization rates vary by age was obtained both within and between studies, allowing for comprehensive comparisons while acknowledging the higher quality of evidence provided by within-study stratifications.

### Quality assessment and meta-analysis

**Fig. 1:**
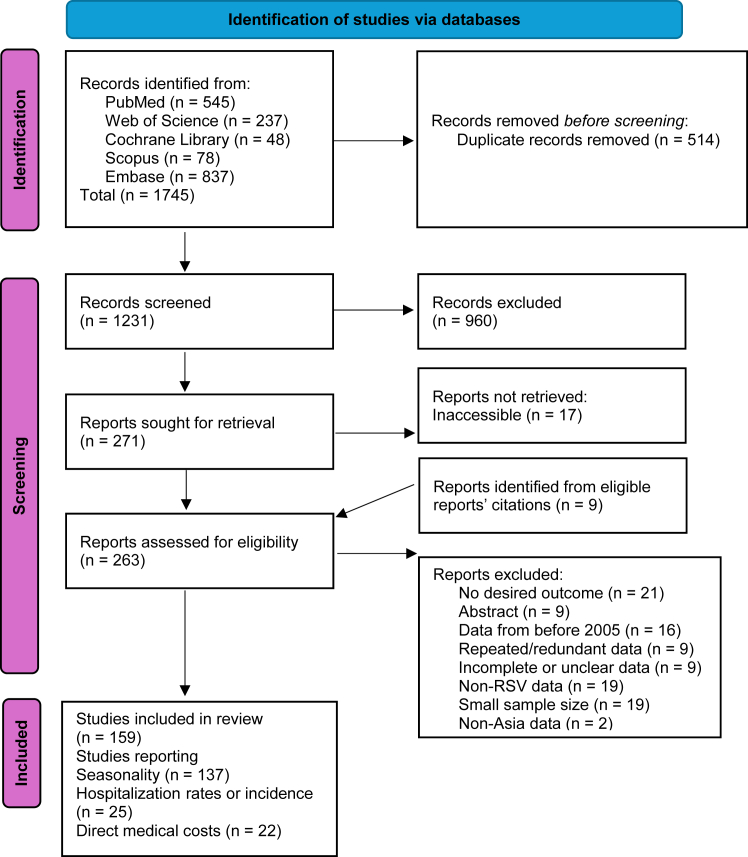
**PRISMA flowchart of the article selection process**.

Articles were screened, analyzed, and visualized in accordance with the Preferred Reporting Items for Systematic Reviews and Meta-Analyses (PRISMA) guidelines (Fig. 1).^23^ Each selected article was critically appraised using an assessment tool tailored to its study design and evaluated outcome (seasonality, hospitalization costs, or incidence). In total, nine different tools were used: for economic evaluation studies (n = 1), for randomized controlled trials (RCTs, n = 1), case–control studies (n = 2), case/time series studies (n = 5), simulation/modeling studies (n = 3), prevalence/incidence surveillance or descriptive observational studies (n = 100), analytical cross-sectional studies (n = 19) (prospective) cohort studies (n = 23), and systematic reviews (n = 5). To remain as consistent as possible, the Joanna Briggs Institute (JBI) appraisal tools were used for all study types except for the simulation/modeling study, which was assessed using a checklist adapted from the transparent reporting of a multivariable prediction model for individual prognosis or diagnosis (TRIPOD).^24^, ^25^, ^26^, ^27^ The primary purpose of this assessment was to evaluate each study's risk of bias (RoB).

However, the risk of bias level assignment was intended for comprehensive and illustrative purposes rather than as a definitive scoring system. Summary scales and aggregate quality scores are not commonly recommended due to several limitations, such as the inability to weigh different study characteristics equally, variability in how scores are calculated, and the potential for introducing bias by producing different results depending on the scoring system used, as summarized in recent literature.^28^ The RoB classification was determined based on the highest RoB across assessed domains.^29^ The main domains evaluated included selection bias, performance bias, detection bias, attrition bias, reporting bias, and other potential sources of bias. Critical appraisal and risk of bias assessments were performed independently by three reviewers (LJP, DJS, TW). To meta-analyze the mean direct medical costs per RSV episode, we used a random-effects model with restricted maximum likelihood (REML) estimation to account for between-study heterogeneity. This approach provided pooled mean estimates along with corresponding estimates of τ^2^ (the variance in effect sizes across studies), I^2^ (the percentage of total variability due to true heterogeneity rather than sampling error), and H^2^ (the relative excess in observed variation). This method follows the framework described by Viechtbauer (2010).^30^ Publication bias was also assessed through a funnel plot, which visually examines the relationship between study size and effect size. In the absence of bias, the plot should resemble a symmetrical inverted funnel, while asymmetry might suggest potential publication bias or small-study effects. Sensitivity analyses were performed for the meta-analysis results by including only those studies with low RoB. The meta-analysis was performed in RStudio Version 2025.05.1 + 513.

### Role of funding source

Jue Tao Lim was supported by the Lee Kong Chian School of Medicine—Ministry of Education Start-Up Grant. The funders had no role in study design, data collection and analysis, decision to publish, or preparation of the manuscript.

## Results

Combined search results produced a total of 1745 articles, of which 1242 were screened by title and abstract for eligibility after removing duplicates. 280 of those articles were retrieved for full-text assessment, and it was determined that 159 met all the inclusion criteria.^6^^,^^9^^,^^10^^,^^31^, ^32^, ^33^, ^34^, ^35^, ^36^, ^37^, ^38^, ^39^, ^40^, ^41^, ^42^, ^43^, ^44^, ^45^, ^46^, ^47^, ^48^, ^49^, ^50^^,^^51^, ^52^, ^53^, ^54^, ^55^, ^56^, ^57^, ^58^, ^59^, ^60^, ^61^, ^62^, ^63^, ^64^, ^65^, ^66^, ^67^, ^68^, ^69^, ^70^, ^71^, ^72^, ^73^, ^74^, ^75^, ^76^, ^77^, ^78^, ^79^, ^80^, ^81^, ^82^, ^83^, ^84^, ^85^, ^86^, ^87^, ^88^, ^89^, ^90^^,^^91^, ^92^, ^93^, ^94^, ^95^, ^96^, ^97^, ^98^, ^99^, ^100^, ^101^, ^102^, ^103^, ^104^, ^105^, ^106^, ^107^, ^108^^,^^109^, ^110^, ^111^, ^112^, ^113^, ^114^, ^115^, ^116^, ^117^, ^118^, ^119^, ^120^, ^121^, ^122^, ^123^, ^124^, ^125^, ^126^, ^127^, ^128^, ^129^, ^130^, ^131^, ^132^, ^133^, ^134^, ^135^, ^136^, ^137^, ^138^^,^^139^, ^140^, ^141^, ^142^, ^143^, ^144^, ^145^, ^146^, ^147^, ^148^, ^149^, ^150^, ^151^, ^152^, ^153^, ^154^, ^155^, ^156^, ^157^, ^158^, ^159^, ^160^, ^161^^,^^162^, ^163^, ^164^, ^165^, ^166^, ^167^, ^168^, ^169^, ^170^, ^171^, ^172^, ^173^, ^174^, ^175^, ^176^, ^177^, ^178^, ^179^, ^180^, ^181^, ^182^, ^183^, ^184^ Of these, 137 studies provided information on seasonality trends, 25 reported RSV hospitalization rates, and 22 included medical costs related to RSV. The included studies spanned a variety of healthcare settings and populations across Asia, encompassing both temperate and tropical climates. RoB was assessed using study design-specific tools, with the JBI checklists applied to most studies and a TRIPOD-adapted checklist used for the simulation/modeling study. Based on this evaluation, 55 (35%) studies were classified as having a “moderate” RoB, while 104 (65%) were classified as “low,” and none were categorized as “high.” Selection bias was commonly observed in studies focusing on specific subpopulations, such as patients with pre-existing conditions (e.g., heart disease or asthma) or those with unclear inclusion criteria. Additionally, many cohort and surveillance studies lacked sufficient information on loss to follow-up/incomplete reporting rates, making attrition bias difficult to assess. Some case–control studies provided limited detail on matching procedures, while several cross-sectional studies had unclear methods for handling missing data. No clear geographic pattern of risk of bias was identified. All checklists and notes regarding risk of bias assessments are included in the Supplementary Materials.

### Seasonality trends

RSV seasonality varied significantly across Asia, as observed among the 137 studies and 28 different countries reported in this SRMA. 53 studies had a moderate RoB (38.7%) and 84 had a low RoB (61.3%). In temperate regions, RSV activity typically peaked during colder months, while in tropical climates, outbreaks coincided with the rainy season or periods of high humidity. In Bangladesh, RSV consistently peaked between September and December, with one observed peak in July. with both studies reporting low RoB.^31^^,^^32^ India showed similar trends, with RSV seasonality aligning with the monsoon (June to September) and winter seasons (October to February), though the timing varied by region.^9^^,^^43^, ^44^, ^45^, ^46^^,^^112^^,^^152^^,^^168^ On the other hand, different studies from China observed peaks in essentially every month of the year.^6^^,^^34^, ^35^, ^36^, ^37^^,^^40^^,^^85^, ^86^, ^87^, ^88^^,^^93^^,^^97^, ^98^, ^99^, ^100^^,^^104^^,^^109^^,^^113^^,^^115^, ^116^, ^117^^,^^119^^,^^120^^,^^122^, ^123^, ^124^, ^125^^,^^130^^,^^136^^,^^138^^,^^144^^,^^145^^,^^147^^,^^149^, ^150^, ^151^^,^^153^^,^^158^^,^^164^^,^^167^^,^^170^^,^^174^^,^^178^^,^^179^^,^^183^

**Fig. 2:**
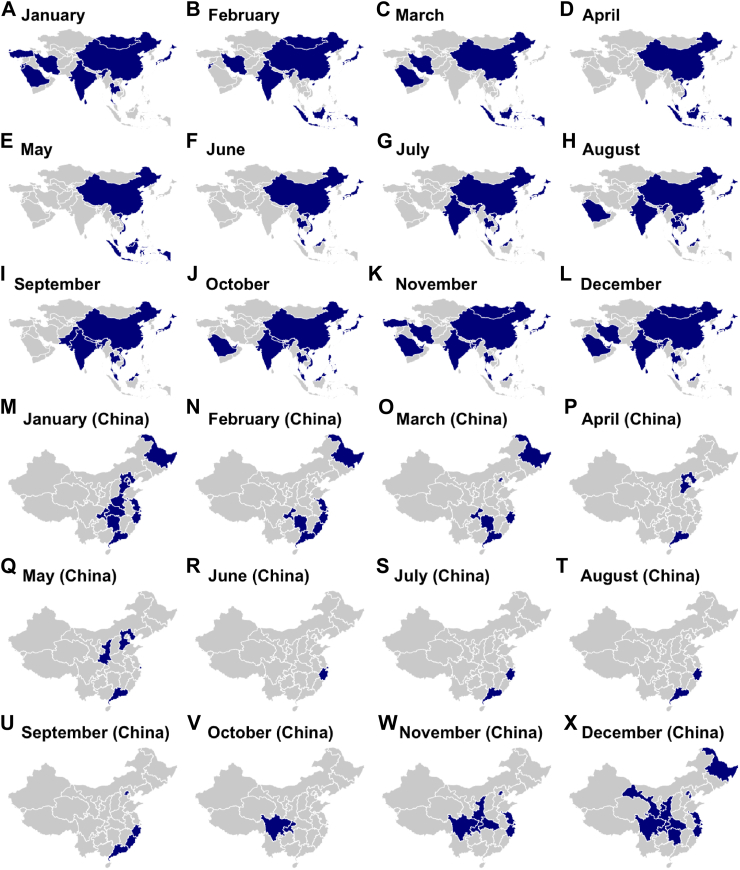
**RSV peaks in Asia by month**. Panels A–L correspond to each month in a calendar year for all Asian countries. Panels M–X correspond to each month in a calendar year for China provinces/municipalities. Regions with a reported peak of RSV cases within the depicted month are shaded in blue, while the gray regions are those without seasonality data.

Tropical countries, including Malaysia, Thailand, and Vietnam, typically observed RSV peaks during the rainy season from May to October, although some studies noted extended activity into December.^9^^,^^62^^,^^78^^,^^80^, ^81^, ^82^^,^^84^^,^^90^^,^^92^^,^^94^^,^^101^, ^102^, ^103^^,^^105^^,^^106^^,^^111^^,^^127^^,^^128^^,^^139^^,^^141^^,^^154^^,^^162^^,^^175^ For instance, RSV activity in Malaysia peaked in July to December, while in Thailand, the peak extended from June to January. Seasonality patterns for all included studies and number of studies that reported RSV seasonality per country are summarized and categorized by country in Table 1, and RSV peaks for each month are depicted on Fig. 2 by country or region.

**Table 1:** RSV seasonality within Asia.

Country (N)	Author (year)	Sample size	Peak RSV activity	RoB
Bangladesh (2)	Homaira et al. (2016)	209	September–December	Moderate
	Haynes et al. (2013)	–	July, October, November	Moderate
Bhutan (1)	Dorji et al. (2024)	248	June and August	Low
China (49)	Feng et al. (2025)	41,706	December–January	Low
	Zhang et al. (2022)	3449	December–February	Low
	Hao et al. (2023)	7861	November–January	Low
	Qiu et al. (2022)	1105	December–January	Low
	Sun et al. (2021)	1451	September and February	Low
	Ye et al. (2016)	3650	December–January	Low
	Yan et al. (2023)	317	No significant seasonal trend	Low
	Zhang et al. (2020)	15,858	December–March	Moderate
	Tian et al. (2017)	3796	December and January	Moderate
	Jin et al. (2012)	331	December	Moderate
	Liu et al. (2019)	1690	January–April and September	Low
	Xiang et al. (2013)	95	January, March, September, and November	Low
	Wang et al. (2018)	56	December–February	Moderate
	Wang et al. (2016)	2971	March and September	Low
	Wang et al. (2025)	6222	December–January	Low
	Yu et al. (2019)	1270	December–January	Low
	Zhang et al. (2009)	103	March	Moderate
	Xu et al. (2021)	4091	January–March	Low
	Xie et al. (2024)	7566	December–March	Low
	Zhao et al. (2025)	7460	January pre-pandemic; August–September post-pandemic	Moderate
	Sun et al. (2025)	7857	December–January pre-pandemic; no trend post-pandemic	Low
	Shi et al. (2024)	10,241	December–January	Low
	Li et al. (2023)	2410	October–March	Moderate
	Li et al. (2022)	5759	December–January	Low
	Zhao et al. (2024)	186	January–March pre-pandemic; July–September post-pandemic	Moderate
	Kuang et al. (2024)	14,564	January–April pre-2017; September post-2017	Low
	Luo et al. (2020)	623	February–April	Moderate
	Wu et al. (2023)	6191	January	Low
	Xu et al. (2025)	300	November–December	Moderate
	Gu et al. (2024)	406	December	Low
	Wei et al. (2024)	216	May	Moderate
	He et al. (2014)	296	March–May and November–December	Moderate
	Li et al. (2025)	112	January and April–May	Low
	Duan et al. (2021)	1452	October–December	Low
	Jiang et al. (2023)	7909	November–January	Low
	Chen et al. (2014)	2508	December–February	Low
	Yu et al. (2024)	112	January	Low
	Lu et al. (2015)	374	December–February	Low
	Li et al. (2024)	273	November–December and May	Low
	Chen et al. (2024)	31,929	November–January and August–September	Moderate
	Ren et al. (2023)	634	January	Moderate
	Ye et al. (2024)	1998	November–February and June	Low
	Li et al. (2013)	87	December	Moderate
	Guo et al. (2024)	194,596	November–March	Low
	Zhang et al. (2013)	171	November–February	Moderate
	Wu et al. (2024)	1501	June–August	Moderate
	Zhang et al. (2015)	81,747	December	Low
	Ren et al. (2022)	4107	December–January	Moderate
	Sun et al. (2022)	1618	February and August	Moderate
Cyprus (1)	Panayiotou et al. (2014)	128	January–February	Low
Hong Kong SAR China (8)	Leung et al. (2014)	118	March	Low
	Chan et al. (2015)	4839	March–April and September	Low
	Tang et al. (2010)	2884	March–May and July–August	Moderate
	Chan et al. (2023)	1816	No clear seasonal pattern	Low
	Ye et al. (2024)	1998	August and December	Low
	Lee et al. (2019)	3538	April and July	Moderate
	Chiu et al. (2010)	100	March–August	Low
	Lam et al. (2019)	–	March–August	Moderate
India (8)	Kini et al. (2019)	94	August–November	Low
	Bhardwaj et al. (2024)	1052	July–September and November–December	Low
	Sabastin et al. (2024)	74	October	Moderate
	Chadha et al. (2020)	1998	July and October	Moderate
	Satav et al. (2021)	315	September and November–January	Low
	Broor et al. (2018)	–	November–February	Moderate
	Saha et al. (2015)	82	September–October and January–February	Low
	Krishnan et al. (2019)	139	September–October	Low
Indonesia (1)	Simões et al. (2011)	163	February–May	Low
Iran (2)	Tavakoli et al. (2021)	74	February–March	Moderate
	Salimi et al. (2016)	775	November–March	Moderate
Israel (2)	Kassem et al. (2019)	309	January	Low
	Weinberger Opek et al. (2021)	430	November and January	Moderate
Japan (10)	Kobayashi et al. (2022)	18,220	September	Low
	Arashiro et al. (2024)	90,413	September, October, and December pre-pandemic	Low
	Nagasawa et al. (2024)	252	July–August	Low
	Otsuka et al. (2025)	505,893	September pre-pandemic; July post-pandemic	Low
	Mizuta et al. (2013)	329	October–January	Moderate
	Ozeki et al. (2022)	945	June–August	Low
	Kume et al. (2022)	639	July–September	Low
	Nagasawa and Ishiwada (2021)	–	December	Low
	Furuta et al. (2018)	1103	September and December	Low
	Lam et al. (2019)	–	August–November	Moderate
Jordan (4)	Yanis et al. (2021)	1397	January–February	Low
	Khuri-Bulos et al. (2010)	467	January–February	Low
	Halasa et al. (2015)	1394	January–February	Low
	Biggs et al. (2023)	358	January	Low
Laos (1)	Nguyen et al. (2017)	157	June–September	Low
Malaysia (7)	Chan et al. (2024)	697	July–August and October–December	Low
	Chan et al. (2023)	435	July–August and October–December	Low
	Khor et al. (2012)	1913	September–December	Moderate
	Toh et al. (2019)	122	June–July (RSV-A) and April–May (RSV-B)	Moderate
	Low et al. (2022)	3652	July–September	Moderate
	Teck et al. (2019)	93	November	Moderate
	Lam et al. (2019)	–	No significant seasonal pattern	Moderate
Mongolia (3)	Do et al. (2024)	2113	December–January	Low
	Chadha et al. (2020)	1803	November–February	Moderate
	Lam et al. (2019)	–	December–January	Low
Myanmar (1)	Phyu et al. (2021)	244	September–October	Moderate
Nepal (2)	Chu et al. (2016)	311	September–October	Moderate
	Mathisen et al. (2011)	88	September–October	Moderate
Pakistan (1)	Ali et al. (2017)	223	September	Low
Qatar (2)	Perez-Lopez et al. (2022)	>828	October–December	Low
	Al-Romaihi et al. (2020)	6102	November–December	Low
Saudi Arabia (3)	Dhayhi et al. (2024)	195	December–January; March in 2017	Low
	Alkharsah et al. (2022)	336	December pre-pandemic; August post-pandemic	Low
	Gashgarey et al. (2024)	885	October–November	Moderate
Singapore (3)	Lee et al. (2023)	15,715	June–August	Low
	Tam et al. (2020)	7691	May–September	Low
	Tan et al. (2020)	9768	August	Low
South Korea (3)	Kim et al. (2018)	1321	October and December	Low
	Lee et al. (2020)	6304	November–December	Moderate
	Kang et al. (2019)	92	October–December	Low
Sri Lanka (1)	Jayaweera et al. (2021)	94	April and December–January	Moderate
Taiwan (3)	Hsu et al. (2014)	3572	April and September	Low
	Chi and Chung (2023)	55,884	February–May and July–August	Low
	Chi et al. (2011)	11,081	March–June and September–December	Low
Thailand (11)	Sitthikarnkha et al. (2022, 1)	19,340	August–October	Low
	Chuaychoo et al. (2019)	69	August & October	Moderate
	Sitthikarnkha et al. (2022, 2)	16,216	August–October	Low
	Naorat et al. (2013)	1137	September–October	Low
	Thongpan et al. (2020)	1082	July–November	Moderate
	Chaiut et al. (2023)	358	September and December–January	Moderate
	Chadha et al. (2020)	3106	July and September	Moderate
	Mahikul et al. (2019)	–	August–October	Low
	Fry et al. (2010)	987	June–October	Low
	Hasan et al. (2014)	1430	June–October	Low
	Chittaganpitch et al. (2018)	643	September–October	Low
The Philippines (2)	Suzuki et al. (2012)	165	October	Moderate
	Biggs et al. (2023)	124	October and December	Low
Turkey (1)	Caglar et al. (2023)	1073	January	Low
United Arab Emirates (2)	Al Shibli et al. (2021)	190	December	Moderate
	Joury et al. (2024)	25,729	October–November	Low
Vietnam (6)	Tuan et al. (2015)	254	August–October	Moderate
	Althouse et al. (2018)	455	August	Low
	Takahashi et al. (2025)	129	April–October	Low
	Tran et al. (2016)	257	October	Moderate
	Yoshihara et al. (2016)	426	August–October	Low
	Do et al. (2016)	302	May–October	Moderate

N: Number of studies included per country.

### Incidence and hospitalization rates of RSV

A total of 25 studies across 16 countries or administrative regions reported on RSV hospitalization or incidence rates. 6 (24%) studies had a moderate RoB and 19 (75%) had a low RoB. Hospitalization rate data were aggregated by age group—infants <6 months of age, infants <12 months of age, children <5 years of age, individuals ≥65 years of age, and study populations inclusive of multiple age groups—as well as by clinical syndrome classification—acute respiratory (tract) infection, acute lower respiratory infection, lower respiratory tract infection, and severe lower respiratory tract infection (Table 2). RSV hospitalization rates were highest among infants and young children, particularly those under 1 year of age, who bore the greatest burden. In Singapore, hospitalization rates among the general population of infants under 6 months were reported at 33.5 per 1000 children per year, based on a study including all RSV-associated acute respiratory tract infections (ARTIs) requiring hospitalization.^72^ Similarly, in Hong Kong, hospitalization rates for the same age group ranged from 23.3 to 31.1 per 1000 children per year.^41^ For studies reporting rates for all children under 5 years (including infants), hospitalization rates varied widely across regions. In China, rates were approximately 14 per 1000 children per year, while in the Philippines, rates reached as high as 62 per 1000 children per year for severe lower respiratory tract infections (LRTIs) associated with RSV.^35^^,^^67^ Many of these findings were supported by within-study age-specific data, which provide stronger internal validity.^36^^,^^45^, ^46^, ^47^^,^^56^^,^^64^^,^^67^^,^^72^^,^^74^^,^^77^^,^^141^^,^^186^ Similarly, RSV hospitalization rates among the elderly are notable. One study reported a hospitalization rate of 1.3 per 1000 persons per year in individuals aged over 65 years in Thailand, while another found a hospitalization rate of 0.42 per 1000 persons per year in the same age group.^81^^,^^141^

**Table 2:** RSV hospitalization rates in Asia.

Population group	Hospitalization rates of RSV-related clinical syndrome classifications (per 1000 person-years)			
	RSV-ARI/ARTI	RSV-ALRI	RSV-LRTI	Severe RSV-LRTI
Infants (<6 months)	0.6–33.5^42^^,^^46^^,^^56^^,^^72^^,^^77^^,^^185^	70^36^	–	–
Infants (<1 year)	2.7–83.1^46^^,^^56^^,^^64^^,^^141^^,^^186^	0.03^44^	51.5–124.0^67^	–
Children (<5 years)	0.5–15.8^31^^,^^45^^,^^56^^,^^68^^,^^72^^,^^77^^,^^81^^,^^140^^,^^141^^,^^165^	14^36^	62.1^67^	22.1^67^
Elderly (≥65 years)	0.42–1.3^81^^,^^141^	–	–	–
General population (All ages)	0.3–2.7^64^^,^^68^^,^^76^	0.31–8.5^78^^,^^141^	–	–

RSV-ALRI: acute lower respiratory infection caused by RSV.

RSV-ARI: acute respiratory infection caused by RSV.

RSV-ARTI: acute respiratory tract infection caused by RSV. Interchangeable with ARI.

RSV-LRTI: lower respiratory tract infection caused by RSV.

Severe LRTI: severe lower respiratory tract infection requiring intensive care.

Going beyond hospitalization rates, overall RSV incidence reported from community-based studies in India showed an incidence rate of 26.2 per 1000 children per year for late preterm infants compared to 14.1 per 1000 children per year for full-term infants, indicating that gestational age significantly influences RSV susceptibility.^45^ Studies in Nepal reported overall RSV incidence of 213 per 1000 persons per year in the general population, with rates spiking to 443 per 1000 persons per year during epidemic periods.^64^ All incidence and hospitalization rates, along with subgroup definitions, are detailed in Table S2.

### Healthcare costs

**Fig. 3:**
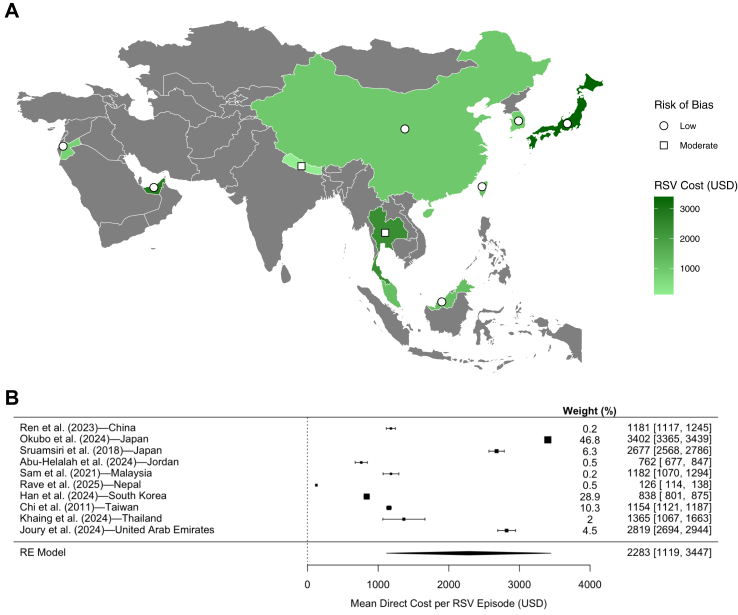
**Mean direct medical cost per RSV episode by country within Asia**. **(A)** Countries are shaded in based on their mean RSV direct medical cost per episode (US$), with darker shades indicating higher costs and grey representing missing data. RoB is indicated by squares for moderate risk and circles for low. **(B)** Meta-analysis results using weighted mean direct medical costs. For individual countries, their mean costs and 95% CIs are displayed. The pooled estimate depicts the pooled effect size (mean cost) and 95% confidence intervals. The square size is according to the weight of each study, based on its sample size and standard error (SE). All costs were rounded to the nearest US dollar.

The economic burden of RSV-related hospitalizations varied widely across the 22 studies and 10 countries or regions with reported relevant data found in this review. 7 (32%) studies had a moderate RoB and 15 (68%) had a low RoB. In mainland China, an upper middle-income country, the mean direct medical cost per RSV episode ranged from US$770–1181.^38^^,^^39^^,^^179^ Studies from Japan reported higher hospitalization costs, with a mean ranging from US$2677–3402 per episode.^51^^,^^55^ In Southeast Asia, RSV-related direct medical costs were generally lower. Mean direct medical costs were US$1182 and US$1365 in Malaysia, and Thailand, respectively.^61^^,^^157^ In Thailand, non-hospitalized patients incurred significantly lower costs—the mean cost for both inpatients and outpatients is US$1365 whereas the inpatient-only cost is US$2448 per RSV episode.^157^ All reported costs and their respective study details are outlined in Table 3. Countries with reported mean direct medical costs are illustrated in Fig. 3A. If multiple studies reported differing costs for the same country, the study with most recent data was used for illustration purposes.

**Table 3:** RSV-related mean direct medical costs in Asia.

Summary statistic	Country	Reference	Sample size	Direct medical cost	95% CI/IQR	RoB
Mean	China	Sun et al. (2025)^a^	7857	$770.41	–	Low
		Ren et al. (2023)^a^	170	$1181.03	$1117.13–$1245.05	Moderate
		Zhang et al. (2014)^a^	959	$812.98	–	Moderate
	Japan	Okubo et al. (2024)^a^	2018: 12,121	$3302.80	$3269.24–$3336.37	Low
			2019: 15,861	$3321.48	$3288.83–$3354.13	
			2020: 2104	$3478.60	$3366.11–$3591.09	
			2021: 14,441	$3477.79	$3449.10–$3506.48	
			2022: 5947	$3608.42	$3560.16–$3656.68	
			Total: 50,474	$3402.07	$3365.17–$3438.98	
		Sruamsiri et al. (2018)^a^	6811	$2677.10	$2567.75–$2786.45	Low
	Jordan	Abu-Helalah et al. (2024)^a^	506	$762.32	$676.69–$847.95	Low
		Khuri-Bulos et al. (2010)^a^	467	$1030.64	–	Low
	Malaysia	Sam et al. (2021)^a^	200	$1182.25	$1070.17–$1294.33	Low
	Nepal	Rave et al. (2025)	496	$125.95	$113.97–$137.93	Moderate
	South Korea	Han et al. (2024)	31,206	Overall: $838.23	$801.34–$875.12	Low
			22,031	Inpatient: $833.10	$823.88–843.35	
	Taiwan	Chi et al. (2011)^a^	11,081	$1154.14	$1120.71–$1187.57	Low
	Thailand	Khaing et al. (2024)	2122	Overall: $1365.35	$1067.08–$1663.62	Low
			1097	Inpatient: $2447.56	$2032.32–$2862.80	
	United Arab Emirates	Joury et al. (2024)^a^	4849	$2819.36	$2693.90–$2944.82	Low
Median	China	Li et al. (2024)^a^	2410	$1163.69	$915.85, $1569.11	Low
		Feng et al. (2025)^a^	41,706	$982.80	$718.04, $1422.98	Low
		Yu et al. (2024)^a^	112	$1006.92	$800.52, $1338.95	Low
		Xie et al. (2024)^a^	7566	$1016.53	$818.76, $1395.68	Low
		Sun et al. (2025)^a^	7857	$611.91	$461.60, $851.54	Low
		Zhang et al. (2014)^a^	959	$815.19	$633.61, $1052.57	Moderate
		Sun et al. (2022)^a^	1618	$1085.28	–	Moderate
	Japan	Inoue et al. (2025)	1025	$2232.08	$1625.93, $3073.57	Low
		Sruamsiri et al. (2018)^a^	6811	$1977.06	$1536.19, $2570.13	Low
	Malaysia	Sam et al. (2021)^a^	200	$990.89	$739.23, $1293.66	Low
	Nepal	Rave et al. (2025)	496	$106.17	$80.22, $142.56	Moderate
	South Korea	Han et al. (2024)	Overall: 31,206	$395.55	$27.67, $805.44	Low
			Inpatient: 22,031	$709.11	$514.41, $995.01	
		Yoon et al. (2020)^a^	118	$3553.94	$1944.03, $6695.92	Moderate
	Thailand	Khaing et al. (2024)	Overall: 2122	$302.17	$74.98, $947.91	Low
			Inpatient: 1097	$813.61	$483.47, $1517.56	
		Tan et al. (2023)	Overall: 1370	$603.22	$186.90, $2356.91	Moderate
			Inpatient: 683	$2362.51	$1543.30, $3561.11	
	Vietnam	Do et al. (2023)	Overall: 105	Overall: $63.24	$43.29, $93.37	Moderate
			Inpatient: 84	Inpatient: $71.45	$49.38, $107.17	

All costs in foreign currencies were standardized to United States Dollars (US Dollars) and standardized to end of 2024 inflation-adjusted prices according to the inflation indices reported by the World Bank. For rows reporting mean costs, the corresponding 95% confidence intervals (CIs) are presented in the final column as ‘2.5%–97.5%’. For rows reporting median costs, their interquartile ranges (IQRs) are shown as ‘quartile 1 (Q1), quartile 3 (Q3)’.

a The costs reported for these studies were specifically for inpatients only. All other costs were for inpatients and outpatients combined.

The meta-analysis included a total of 10 studies (and 9 countries or regions) with reported mean direct medical costs and their respective 95% confidence intervals. Studies from which confidence intervals could not be obtained were excluded from the menta-analysis. The pooled mean direct medical cost per RSV-associated hospitalization was estimated at US$2283 (95% CI: US$1119–3447) using a random-effects meta-analysis with restricted maximum likelihood (REML) estimation. Between-study heterogeneity was substantial, with τ^2^ = 1,103,980, I^2^ = 99.96%, and H^2^ = 2288.9, indicating that nearly all observed variability was due to true differences across studies rather than sampling error. Meta-analysis results are presented in Fig. 3B. All costs in the meta-analysis were rounded to the nearest dollar. Sensitivity analysis of including only studies with a low RoB revealed no significant difference with the overall meta-analysis results.

The overall quality of studies included in this review was moderate, with most seasonality and incidence studies rated as low RoB. However, studies on healthcare costs showed greater variability, with several classified as moderate RoB due to inconsistent reporting and methodological weaknesses. Bias from sequence generation was the most commonly reported issue, particularly in studies that focused on narrow subpopulations or lacked clear inclusion criteria.

**Fig. 4:**
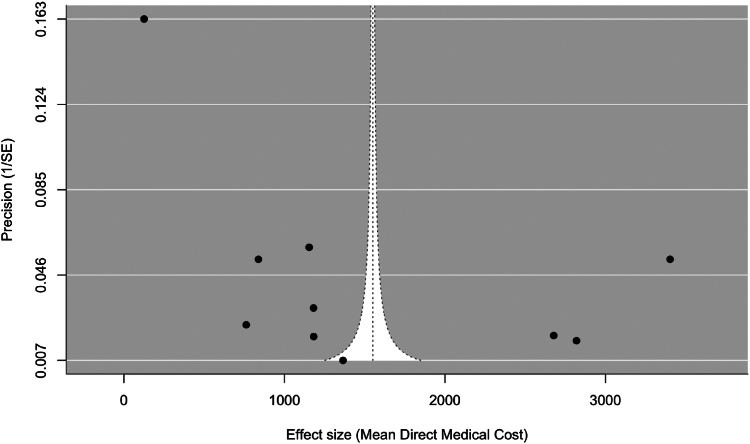
**Funnel plot representation of publication bias among direct medical cost studies in Asia**. Individual study effect sizes (mean direct medical costs) are plotted as points along the x-axis and their precision (inverse of standard error) on the y-axis. The vertical line represents the pooled mean estimate, and the dotted lines delineating the funnel shape indicate the expected 95% confidence region in the absence of bias.

The funnel plot, with precision (1/standard error) on the y-axis, showed asymmetry, with most studies clustered toward the bottom and falling outside the funnel boundaries. Additionally, there was a greater concentration of studies on the left side of the plot, suggesting potential publication bias and/or small-study effects (Fig. 4).

## Discussion

This systematic review highlights the considerable burden of RSV across Asia, revealing significant variability in seasonality, incidence, and healthcare costs, alongside critical gaps in data quality and surveillance. The findings underscore the pressing need for region-specific public health interventions and enhanced epidemiological research to better understand and mitigate the impact of RSV, particularly in LMICs where the burden is often highest and least documented.

Seasonality trends across the region reflect stark differences influenced by climatic and environmental factors. While temperate countries such as China and Japan experience well-defined RSV peaks during winter months, tropical regions such as Thailand, Vietnam, and Malaysia exhibit prolonged outbreaks coinciding with the rainy season.^6^^,^^34^, ^35^, ^36^, ^37^^,^^40^^,^^51^^,^^52^^,^^62^^,^^78^^,^^80^, ^81^, ^82^^,^^84^^,^^141^ These extended or less predictable patterns pose challenges for the timing of interventions such as maternal immunization or potential pediatric vaccine programs. The cyclical nature of RSV in tropical climates, for example, highlights the need for continuous surveillance to determine optimal vaccination windows. Studies from these regions often reported unclear risk of bias due to limited methodological details, emphasizing the need for more rigorous and standardized approaches to data collection.^32^^,^^43^^,^^48^^,^^56^^,^^66^^,^^73^^,^^78^^,^^80^^,^^82^^,^^84^

The burden of RSV is disproportionately borne by infants and young children, particularly those under one year of age even among high-income regions, with hospitalization rates reaching up to 31.1 per 1000 children per year in Hong Kong and 33.5 per 1000 child-years in Singapore.^41^^,^^72^ Findings of higher hospitalization rates among children are consistent across studies and reaffirm the vulnerability of these populations to RSV, highlighting them as priority groups for preventive strategies. Hospitalization rates among children under five years of age vary widely, from 2.32 per 1000 children per year in a high-income country such as Taiwan, to 34 per 1000 children per year in a lower-income country such as Indonesia, demonstrating the variability in regional RSV burden.^47^^,^^77^

General population studies showed lower incidence rates overall but pointed out peaks during epidemic periods, as in Nepal, where RSV incidence surged to 443 per 1000 person-years during outbreaks.^64^ These figures likely underestimate the true burden of RSV in many resource-limited settings, where diagnostic practices are insufficient to capture the full scope of disease.

The economic burden of RSV hospitalizations is substantial and varied widely across Asia, reflecting differences in healthcare systems and resource availability. Southeast Asia exhibited comparatively lower costs, with median direct medical expenses reported at US$63 in Vietnam and US$991 in Malaysia.^61^^,^^83^ However, the relative burden of these costs is likely much higher in resource-limited settings, where healthcare expenses are more often paid out-of-pocket.

Publication bias analysis of studies that measure mean direct medical costs suggests that there may be some publication bias or small-study effects, indicating a tendency to report lower-cost estimates in studies with smaller sample sizes. This may be due to LMICs having fewer resources to conduct large-scale studies the way high-income countries do. These findings reveal the critical need to accurately assess location-specific burdens caused by RSV, and consequently inform resource allocation, especially in low-income settings.

Existing literature supports the findings of this review, with previous systematic reviews confirming that RSV seasonality varies by weather patterns, geographic location, and altitude, aligning with the trends identified in this study.^185^^,^^187^ Similarly, observed incidence patterns reinforce the heterogeneity of RSV transmission dynamics across Asia.^185^ While the burden of RSV is predominantly discussed in the context of pediatric populations, evidence suggests that RSV also poses a significant risk to older adults, particularly those with underlying conditions. Across the continent of Asia, the proportion of RSV-related acute respiratory infections among older patients ranged from 0.2% to 5.6%, while RSV accounted for 1.1%–10.3% of all community-acquired pneumonia cases.^186^ Seasonal variation in RSV incidence among adult inpatients has been reported in Taiwan, with rates reaching approximately 2% during peak periods.^188^ Beyond Asia, RSV burden among elderly populations have been estimated to be as high as 5.2 million cases, 470,000 hospitalizations, and 33,000 inpatient deaths in high-income countries, indicating the significant, but often overlooked, burden of RSV in this population.^189^ Additionally, the long-term financial impact of RSV extends beyond hospitalization, as evidenced by a 2023 study from the United States, which reported total all-cause costs to be US$7797 higher per patient following RSV infection.^190^ These findings emphasize the need for comprehensive RSV prevention strategies that not only prioritize infants and young children but also address the burden in older adults, ensuring that future vaccination and treatment policies reflect the full spectrum of RSV-related morbidity and healthcare costs.

This study has some limitations. First, there was considerable variability across studies in terms of the populations included, the units of observed outcomes, and the surveillance systems used to monitor RSV, which makes it more difficult to accurately characterize RSV seasonality and burden in certain regions. Second, this study only included published articles and did not account for data from surveillance bulletins, which are also important contributors to capture a fuller picture of RSV's impact on public health. Third, the analysis lacks data from several Asian countries, including those in Central Asia (Kazakhstan, Kyrgyzstan, Tajikistan, Turkmenistan, Uzbekistan), East Asia (Macau SAR China, North Korea), South Asia (Afghanistan, Bhutan, Maldives), Southeast Asia (Brunei, Timor-Leste), and especially West Asia (Armenia, Azerbaijan, Bahrain, Cyprus, Georgia, Iraq, Kuwait, Lebanon, Oman, Palestine, Syria, Turkey, Yemen). Fourth, due to a relatively limited number of studies reporting RSV hospitalization or incidence rates, we did not discriminate between those studies performed year-round and those performed during RSV epidemics. As a result, certain studies may have inflated hospitalization rates compared to the studies that took measurements year-round. Finally, the meta-analysis of hospitalization costs showed substantial heterogeneity between studies and settings, limiting the interpretability of pooled estimates. Although we retained the analysis to provide a descriptive summary of reported mean costs, the resulting effect size should be interpreted with caution. Addressing these knowledge gaps will require further research efforts to provide a more comprehensive understanding of RSV burden.

Moving forward, more research is needed to assess the feasibility and impact of RSV interventions in Asia, particularly the implementation of vaccination programs. Modeling studies from Japan in 2024 suggest that RSVPreF3 OA vaccination could prevent over 1,008,000 RSV-ARI cases over three years, demonstrating the potential for vaccines to significantly reduce RSV morbidity and mortality.^189^ Furthermore, we need more information to tackle the lack of serology and surveillance data of the different RSV strains. The findings of this study provide a crucial foundation for decision-making as new RSV vaccines advance through clinical trials and are considered for introduction in the Asia–Pacific region. By addressing data gaps and building on existing knowledge, future efforts can optimize strategies to reduce RSV burden and improve public health outcomes in diverse populations.

Overall, this review reveals a diverse and complex landscape of RSV burden across Asia, accentuating the critical need for tailored public health interventions. Variability in seasonality, disease burden, and economic costs across countries suggest that strengthening regional surveillance systems to provide timely and accurate data is ideal. Enhanced investment in diagnostics, coupled with recent advances in vaccine development, offers an opportunity to reduce RSV-associated morbidity, mortality, and economic burden in this region. These findings provide a robust foundation for future public health planning, with the potential to inform evidence-based policy and resource allocation to address one of the leading causes of pediatric respiratory infections worldwide.

## Contributors

LJP, TW, and DJS: systematic literature search and review, accessed and verified underlying data, data analysis, data interpretation, quality assessment, writing—original draft, writing—review & editing. JYC: systematic literature search, data collection, supervision, writing—review & editing. LEW, PYC, DCBL: project administration, resources, supervision, writing—review & editing. YSL: conceptualization, project administration, writing—review & editing. JTL: conceptualization, methodology, project administration, supervision, writing—original draft, writing—review & editing.

## Data sharing statement

All data utilized or synthesized to support the findings in this study are available within this manuscript its Supplementary Information files.

## Editor note

The Lancet Group takes a neutral position with respect to territorial claims in published maps and institutional affiliations.

## Declaration of interests

The authors declare no conflicts of interest.
