# Supplementary material for: Respiratory syncytial virus hospitalization costs, rates, and seasonality in Asia: a systematic review and meta-analysis
Source: eClinicalMedicine. 2025 Jul 10;86:103350. doi: 10.1016/j.eclinm.2025.103350 (PMC12275161; doi:10.1016/j.eclinm.2025.103350)
Supplement: Supplementary Materials 1 [file mmc1.docx]

**Supplementary Information**

**Respiratory Syncytial Virus Hospitalization Costs, Rates, and Seasonality in Asia: A Systematic Review and Meta-Analysis**

Table of Contents

[Table S1. Summary of Studies Included in this Review. 2](#_Toc199248369)

[Table S2. Hospitalization and Incidence Rates of Studies Reviewed. 8](#_Toc199248370)

[Appendix A. Full Search Terms Among 5 Databases. 11](#_Toc199248371)

[Appendix B. List of Studies Excluded After Full-text Review 13](#_Toc199248372)

# Table S1. Summary of Studies Included in this Review.

| Author | Study Design | Study Year(s) | Country | Measurement(s) | Risk of Bias (RoB) |
| --- | --- | --- | --- | --- | --- |
| Homaira et al (2016) | Cohort and cross-sectional | 2010-2014 | Bangladesh | Incidence, seasonality | Moderate |
| Haynes et al (2013) | Cross-sectional | 2004-2012 | Bangladesh | Seasonality | Moderate |
| Saha et al (2018) | Cohort | 2011-2014 | India | Incidence | Low |
| Zhang et al (2015) | Systematic Review and Meta-Analysis | 2010-2015 | China | Seasonality | Low |
| Sun et al (2022) | Case series | 2014-2017 | China | Seasonality, costs | Moderate |
| Ren et al (2022) | Case series | 2010-2014 | China | Seasonality, incidence | Moderate |
| Zhang et al (2013) | Cohort | 2007-2010 | China | Seasonality | Moderate |
| Guo et al (2024) | Systematic Review | 1993-2019 | China | Seasonality | Low |
| Zhang et al (2014) | Cohort | 2005-2009 | China | Costs | Moderate |
| Ren et al (2023) | Case series | 2020-2021 | China | Costs | Moderate |
| Wu et al (2024) | Cross-sectional | 2022-2024 | China and | Seasonality | Moderate |
| Lee et al (2019) | Case-control | 2013-2015 | Hong Kong SAR China and | Seasonality | Moderate |
| Chiu et al (2010) | Surveillance | 2003-2006 | Hong Kong SAR China | Seasonality, incidence | Low |
| Broor et al (2018) | Systematic Review | 1971-2018 | India | Seasonality | Moderate |
| Krishnan et al (2019) | Cohort and case-control | 2012-2014 | India | Seasonality, incidence | Low |
| Saha et al (2015) | Cross-sectional | 2000-2013 | India | Seasonality, incidence | Low |
| Satav et al (2021) | Cohort | 2016-2020 | India | Seasonality, incidence | Low |
| Simoes et al (2011) | Cohort | 1999-2001 | Indonesia | Seasonality, incidence | Low |
| Tavakoli et al (2021) | Cross-sectional | 2018-2019 | Iran | Seasonality | Moderate |
| Salimi et al (2016) | Systematic Review | 2007-2013 | Iran | Seasonality | Moderate |
| Okubo et al (2024) | Cohort | 2018-2022 | Japan | Costs | Low |
| Furuta et al (2018) | Cohort | 2011-2014 | Japan | Seasonality | Low |
| Nagasawa & Ishiwada (2022) | Systematic Review | 2010-2020 | Japan | Seasonality, | Low |
| Simoes et al (2024) | Cohort | 2011-2017 | Japan | incidence, costs | Moderate |
| Sruamsiri et al (2018) | Case series | 2014-2015 | Japan | Incidence | Low |
| Khuri-Bulos et al (2010) | Cohort | 2007 | Jordan | Costs | Low |
| Halasa et al (2015) | Cohort | 2010-2013 | Jordan | Seasonality, costs | Low |
| Yoon et al (2020) | Cohort | 2012-2015 | South Korea | Seasonality, incidence | Moderate |
| Kang et al (2019) | Cohort | 2008-2013 | South Korea | Costs | Low |
| Lee et al (2020) | Case series | 2010-2015 | South Korea | Seasonality, costs | Moderate |
| Nguyen et al (2017) | Cohort | 2013-2014 | Laos | Seasonality | Low |
| Sam et al (2021) | Cross-sectional | 2013-2015 | Malaysia | Seasonality | Low |
| Low et al (2022) | Surveillance | 2015-2019 | Malaysia | Costs | Low |
| Chaw et al (2016) | Cohort | 2013-2015 | Mongolia | Seasonality, costs | Low |
| Chu et al (2016) | RCT | 2011-2014 | Nepal | Incidence | Moderate |
| Mathisen et al (2011) | Cohort | 2006-2008 | Nepal | Seasonality, incidence, costs | Moderate |
| Ali et al (2017) | Cohort | 2010-2012 | Pakistan | Seasonality | Low |
| Ueno et al (2019) | Cohort | 2014-2016 | The Philippines | Seasonality | Low |
| Kamigaki et al (2017) | Cross-sectional | 2012-2014 | The Philippines | Incidence | Low |
| Al-Romaihi et al (2020) | Surveillance | 2012-2017 | Qatar | Incidence | Low |
| Gashgarey et al (2024) | Cohort | 2019-2022 | Saudi Arabia | Seasonality | Moderate |
| Alharbi et al (2024) | Economic evaluation | 2016-2021 | Saudi Arabia | Seasonality | Low |
| Tam et al (2020) | Cross-sectional | 2005-2014 | Singapore | Costs | Low |
| Lee et al (2023) | Cross-sectional | 2009-2019 | Singapore | Seasonality, incidence, costs | Low |
| Yeo et al (2018) | Cohort | 2005-2015 | Singapore | Seasonality | Low |
| Tan et al (2020) | Cross-sectional | 2011-2016 | Singapore | Incidence | Low |
| Jayaweera et al (2021) | Cohort | 2013-2014 | Sri Lanka | Seasonality | Moderate |
| Chi et al (2011) | Cross-sectional | 2004-2007 | Taiwan | Seasonality, incidence | Low |
| Mahikul et al (2019) | Simulation/modeling | 1995-2011 | Thailand | Seasonality, incidence, costs | Low |
| Tan et al (2023) | Cohort | 2014-2021 | Thailand | Seasonality, incidence | Moderate |
| Hasan et al (2014) | Surveillance | 2005-2010 | Thailand | Costs | Low |
| Fry et al (2010) | Surveillance | 2003-2007 | Thailand | Seasonality | Low |
| Chittaganpitch et al (2018) | Surveillance | 2010-2014 | Thailand | Seasonality, incidence | Low |
| Do et al (2023) | Cohort | 2019-2021 | Vietnam | Seasonality | Moderate |
| Do et al (2016) | Surveillance | 2009-2010 | Vietnam | Costs | Moderate |
| He Ying (2014) | Cohort, Surveillance | July 2007 to June 2010 | China | Seasonality | Moderate |
| Wu et al (2023) | Cross-sectional | 2018-2022 | China | Seasonality | Low |
| Chen et al (2024) | Case series, Surveillance | 2019-2023 | China | Seasonality | Moderate |
| Jiang et al (2023) | Cohort, Surveillance | 2017-2018 | China | Seasonality | Low |
| Rave et al (2025) | Cohort, Surveillance | July-Nov 2023 | Nepal | Seasonality | Moderate |
| Chan et al (2024) | Cross-sectional | 2017-2022 | Malaysia | Direct cost | Low |
| Chen et al (2014) | Surveillance | 2001-2011 | China | Seasonality, Incidence | Low |
| Yoshihara et al (2016) | Surveillance, Cross-sectional | 2010-2012 | Vietnam | Seasonality | Low |
| Li et al (2022) | Surveillance | January 2012–January 2021 | China | Seasonality, Incidence | Low |
| Sitthikarnkha et al (2022) | Surveillance | Oct 2015 - Sept 2020 | Thailand | Seasonality | Low |
| Li et al (2024) | Cross-sectional | 2009-2019 | China | Seasonality | Low |
| Ozeki et al (2022) | Surveillance | 2011-2019 | Japan | Direct cost | Low |
| Ye et al (2024) | Surveillance | 2013-2023 | China and Hong Kong SAR | Seasonality | Low |
| Kuang et al (2024) | Surveillance | 2012-2023 | China | Seasonality | Low |
| Wei et al (2024) | Surveillance | July 2022 - December 2023 | China | Seasonality | Moderate |
| Duan et al (2021) | Surveillance | March 2018 - March 2020 | China | Seasonality | Low |
| Chaiut et al (2023) | Surveillance | 2016-2021 | Thailand | Seasonality | Moderate |
| Tuan et al. (2015) | Surveillance | 2010 | Vietnam | Seasonality, Incidence | Moderate |
| Tran et al (2016) | Surveillance | Apr 2010 - May 2011 | Vietnam | Seasonality | Moderate |
| Feng et al (2025) | Surveillance | 2016-2022 | China | Seasonality | Low |
| Chuaychoo et al (2019) | Cohort, Surveillance | May 2014 - Dec 2015 | Thailand | Seasonality, Direct cost | Moderate |
| Okubo et al (2024) | Cohort, Cross-sectional | 2018-2022 | Japan | Seasonality | Low |
| Takahashi et al (2025) | Cohort, Surveillance | Oct 2009 - Sept 2012 | Vietnam | Direct cost | Low |
| Lam et al (2019) | Surveillance | 2010-2015 | Mongolia, Malaysia, Hong Kong SAR, Japan | Seasonality, Incidence | Moderate |
| Yu et al (2024) | Surveillance | Dec 2023 - May 2024 | China | Seasonality | Low |
| Kassem et al (2019) | Surveillance | 2008-2011 | Israel | Direct cost, Seasonality | Low |
| Li et al (2023) | Surveillance | June 2009 to May 2019 | China | Seasonality | Moderate |
| Weinberger Opek et al (2021) | Surveillance | Jan 2018 - June 2021 | Israel | Seasonality, Incidence | Moderate |
| Chan et al (2023) | Surveillance | 2017-2021 | Malaysia | Seasonality, Incidence | Low |
| Sabastin et al (2024) | Cross-sectional | March 2020-Feb 2022 | India | Seasonality, Incidence | Moderate |
| Li et al (2013) | Surveillance | 2010 | China | Seasonality | Moderate |
| Kume et al (2022) | Surveillance | Sept 2017 - March 2020 | Japan | Seasonality | Low |
| Ren et al (2023) | Case series, Surveillance | 2018-2021 | China | Seasonality | Low |
| Zhang et al (2022) | Surveillance | 2014-2018 | China | Seasonality, Incidence | Low |
| Gu et al (2024) | Surveillance | July 2014 - Dec 2023 | China | Seasonality | Low |
| Lu et al (2015) | Surveillance | 2010-2014 | China | Seasonality | Low |
| Xu et al (2025) | Surveillance | 2022-2023 | China | Seasonality | Moderate |
| Luo et al (2020) | Surveillance | 2011-2016 | China | Seasonality | Moderate |
| Li et al (2024) | Surveillance | June 2021 - May 2023 | China | Seasonality | Low |
| Li et al (2025) | Surveillance | 2021-2023 | China | Seasonality | Low |
| Zhao et al (2024) | Surveillance | Nov 2020 - June 2022 | China | Seasonality | Moderate |
| Hao et al. (2023) | Surveillance | 2018-2022 | China | Seasonality | Low |
| Qiu et al. (2022) | Surveillance | 2019-2021 | China | Seasonality | Low |
| Leung et al. (2014) | Surveillance | Jan 2009 - Jun 2011 | Hong Kong | Seasonality | Low |
| Khor et al. (2012) | Surveillance | 1982-2008 | Malaysia | Seasonality | Moderate |
| Sitthikarnkha et al. (2021) | Surveillance | 2015-2019 | Thailand | Seasonality | Low |
| Al Shibli et al. (2021) | Surveillance | Nov 2008 - Dec 2011 | United Arab Emirates | Seasonality | Moderate |
| Sun et al. (2021) | Surveillance | Oct 2014 - Sept 2017 | China | Seasonality | Low |
| Panayiotou et al. (2014) | Surveillance | Nov 2010 - Mar 2013 | Cyprus | Seasonality | Low |
| Kobayashi et al. (2022) | Surveillance | 2017-2018 | Japan | Seasonality | Low |
| Phyu et al. (2021) | Surveillance | July 2015 - Dec 2018 | Myanmar | Seasonality, Incidence | Moderate |
| Inoue et al. (2025) | Surveillance | Apr 2017 - Mar 2022 | Japan | Seasonality | Low |
| Dhayhi et al. (2024) | Surveillance | 2015-2020 | Saudi Arabia | Direct cost | Low |
| Ye et al. (2016) | Surveillance | 2015 | China | Seasonality | Low |
| Han et al. (2024) | Cohort, Surveillance | Jan 2017 - Apr 2022 | Korea | Seasonality | Low |
| Yan et al. (2023) | Surveillance | 2018-2019 | China | Direct cost | Low |
| Toh et al. (2019) | Surveillance | Jun 2017-May 2018 | Malaysia | Seasonality | Moderate |
| Chan et al. (2015) | Surveillance | 1998-2012 | Hong Kong | Seasonality | Low |
| Naorat et al. (2013) | Surveillance | 2008-2011 | Thailand | Seasonality, Incidence | Low |
| Chadha et al. (2020) | Surveillance | Jan 2017 - Apr 2019 | India, Thailand, Mongolia | Seasonality, Incidence | Moderate |
| Tang et al. (2010) | Simulation/Modeling | May 2000 - Dec 2007 | Hong Kong | Seasonality | Moderate |
| Arashiro et al. (2024) | Surveillance, Cohort | Apr 2011 - July 2022 | Japan | Seasonality | Low |
| Zhang et al. (2020) | Simulation/Modeling | 2008-2017 | China | Seasonality | Moderate |
| Tian et al. (2017) | Surveillance | 2015 | China | Seasonality | Moderate |
| Kim et al. (2018) | Surveillance | 2013-2015 | Korea | Seasonality | Low |
| Jin et al. (2012) | Surveillance | Dec 2006 - Nov 2009 | China | Seasonality | Moderate |
| Shobugawa et al. (2017) | Surveillance | 2007-2014 | Japan | Seasonality | Low |
| Liu et al. (2019) | Cross-sectional, Surveillance | July 2009 - June 2016 | China | Seasonality | Low |
| Xiang et al. (2013) | Surveillance | May 2005 - Apr 2010 | China | Seasonality | Low |
| Wang et al. (2018) | Surveillance | 2014-2017 | China | Seasonality | Moderate |
| Kini et al. (2019) | Surveillance | August 2011-August 2013 | India | Seasonality | Low |
| Wang et al. (2016) | Surveillance | 2012-2015 | China | Seasonality | Low |
| Low et al. (2022) | Surveillance | 2015-2019 | Malaysia | Seasonality | Moderate |
| Teck et al. (2019) | Surveillance | Year not mentioned | Malaysia | Seasonality | Moderate |
| Hsu et al. (2014) | Surveillance, Cohort | Jan 2000 - Aug 2010 | Taiwan | Seasonality | Low |
| Nagasawa et al. (2024) | Cohort, Surveillance | 2011-2016 | Japan | Seasonality | Low |
| Khaing et al. (2024) | Cohort, Surveillance | 2014-2021 | Thailand | Seasonality | Low |
| Wang et al. (2025) | Cohort, Surveillance | 2017-2023 | China | Direct cost | Low |
| Dorji et al. (2024) | Surveillance, Cross-sectional | 2016-2018 | Bhutan | Seasonality | Low |
| Do et al. (2024) | Surveillance | Apr 2015 - June 2021 | Mongolia | Seasonality | Low |
| Otsuka et al. (2025) | Surveillance | 2018-2021 | Japan | Seasonality | Low |
| Thongpan et al. (2020) | Surveillance | 2012-2018 | Thailand | Seasonality | Moderate |
| Chi and Chung (2023) | Surveillance | 2008-2020 | Taiwan | Seasonality | Low |
| Yu et al. (2019) | Surveillance | July 2007–June 2015 | China | Seasonality | Low |
| Yoshida et al. (2013) | Surveillance | Apr 2007 - Mar 2010 | Vietnam | Seasonality | Low |
| Suzuki et al. (2012) | Cohort, Surveillance | May 2008-May 2009 | The Philippines | Incidence | Moderate |
| Zhang et al. (2009) | Surveillance, Cohort | 2008 | China | Seasonality | Moderate |
| Bhardwaj et al (2024) | Surveillance | Jan 2017 - Feb 2023 | India | Seasonality | Low |
| Joury et al. (2024) | Cohort, Surveillance | Jan 2014 - Sept 2023 | Dubai, UAE | Seasonality | Low |
| Xu et al. (2021) | Surveillance | Jan 2009 - Sept 2013 | China | Seasonality, Direct cost | Low |
| Mizuta et al. (2013) | Surveillance | 2004 - 2011 | Japan | Seasonality | Moderate |
| Miyama et al. (2021) | Surveillance | 2012-2019 | Japan | Seasonality | Low |
| Xie et al (2024) | Surveillance | July 2013 - June 2022 | China | Seasonality | Low |
| Althouse et al (2018) | Surveillance | Jan 2007 - Apr 2012 | Vietnam | Seasonality, Direct cost | Low |
| Yanis et al. (2021) | Surveillance | Mar 2010 - Mar 2013 | Jordan | Seasonality | Low |
| Caglar et al. (2023) | Surveillance | Apr 2018 - Mar 2023 | Turkey | Seasonality | Low |
| Zhao et al. (2025) | Cross-sectional | 2019-2023 | China | Seasonality | Moderate |
| Sun et al. (2025) | Cohort, Surveillance | Jan 2018 - Aug 2023 | China | Seasonality | Low |
| Abu-Helalah et al. (2024) | Cross-sectional | Nov 2022 - Apr 2023 | Jordan | Seasonality, Direct cost | Low |
| Alkharsah et al (2022) | Surveillance | Jan 2015 - Feb 2022 | Saudia Arabia | Direct cost | Low |
| Chan et al. (2023) | Surveillance | Jan 2014 - Apr 2023 | Hong Kong | Seasonality | Low |
| Shi et al. (2024) | Surveillance | July 2012 - June 2022 | China | Seasonality | Low |
| Perez-Lopez et al. (2022) | Surveillance | July 2019 - Jan 2022 | Qatar | Seasonality | Low |
| Biggs et al. (2023) | Surveillance | 2015-2017 | Jordan and the Philippines | Seasonality | Low |

# Table S2. Hospitalization and Incidence Rates of Studies Reviewed.

| **Population Group** | **Author (Year)** | **Country** | **Incidence of disease** | **RoB** |
| --- | --- | --- | --- | --- |
| Newborns | Saha et al. (2018) | Bangladesh, India, and Pakistan | RSV total incidence: 5.4 (95% CI 4.8–6.3) per 1000 livebirths.  Incidence >2.5 per 1000 livebirths in all sites, peaking in Sylhet, Bangladesh (7.3 infections per 1000 livebirths) | Low |
| Infants | Chiu et al. (2010) | Hong Kong SAR | Population of infants <6 months, rates of RSV hospitalization were 23.34-31.12 per 1000 children per year | Low |
|  | Satav et al. (2021) | India | Severe RSV and hospitalization rates for children aged:  0–11 months: 22.4 (95% CI: 18.6–27.0); 14.1 (11.1–17.8) per 1000 children per year  Preterm infants: 26.2 (17.8–38.5); 12.6 (7.2–22.0) per 1000 children per year  <6 months: 15.9 (11.8–21.4); 12.9 (9.3–18.0) per 1000 children per year  Preterm <6 months: 26.3 (15.4–45.0); 10.1 (4.2–24.2) per 1000 children per year | Low |
|  | Simões et al. (2024) | Japan | RSV incidence and hospitalization rates in children aged 0-5 months: 1.43 and 0.6 per 1000 children per year  Full-term infants: 1.34 and 0.58 per 1000 children per year  Late preterm infants: 2 and 0.68 per 1000 children per year | Moderate |
|  | Halasa et al. (2015) | Jordan | RSV hospitalization rates in children aged:  <6 months: 21.1–25.9 per 1000 children per year  6-11 months: 6–8 per 1000 children per year  12-23 months: 1.6–2.5 per 1000 children per year | Low |
|  | Tam et al. (2020) | Singapore | Hospitalization rate in children aged:  <6 months: 33.5 per 1000 children per year  6–29 months: 13.2 per 1000 children per year | Low |
|  | Yeo et al. (2018) | Singapore | Incidence rates in children aged:  ≤26 weeks: 65 per 1000 infants per year  <3 months: 75.9 per 1000 infants per year  3 to <6 months: 93.1 per 1000 infants per year  6 months to 1 year: 60.1 per 1000 infants per year | Low |
| Children | Homaira et al. (2016) | Bangladesh | 3 RSV hospitalizations per 1000 children per year | Moderate |
|  | Ren et al. (2022) | China | RSV-ALRI hospitalization rate of children aged:  0–59 months: 14 per 1000 children per year  0–5 months: 70 per 1000 children per year | Moderate |
|  | Saha et al. (2015) | India | RSV–associated hospitalization per 1000 children per year among children aged:  0–5 months: 15.2 (95% CI: 8.3–26.8)  6–23 months: 5.3 (3.2–8.7)  24–59 months: 0.5 (0.1–1.5) | Low |
|  | Krishnan et al. (2019) | India | Adjusted ALRI incidence in RSV episodes: 0.03 (95% CI: 0.02-0.03) per 1000 children per year | Moderate |
|  | Simões et al. (2011) | Indonesia | RSV incidence among children aged:  6-8-months: 103.0 per 1000 children per year  <6 months: 16.5 per 1000 children per year  6-11 months: 83.1 per 1000 children per year  12-23 months: 67.0 per 1000 children per year  2-5-years: 28.1 per 1000 children per year | Low |
|  | Nagasawa and Ishiwada (2021) | Japan | Mean RSV incidence in children aged:  0–4 years: 19.53 per 1000 persons per year | Low |
|  | Ueno et al. (2019) | Philippines | The overall incidence rates of RSV‐LRTI and severe RSV‐LRTI in children aged:  2-59 months: 62.1 and 22.1 per 1000 children per year  2‐23 months: 124.0 and 51.5 per 1000 children per year | Low |
|  | Chi et al. (2011) | Taiwan | Average annual hospitalization incidence for children aged:  <5 years old: 2.32 per 1000 children per year  1-2 months: 16.37 per 1000 children per year | Low |
|  | Yoshida et al. (2013) | Vietnam | RSV-ARI incidence rate of children less than 60 months: 9.59 per 1000 children per year | Low |
| Pregnant women | Chaw et al. (2016) | Mongolia | RSV incidence rate: 109.5 per 1000 persons per year (95% C.I: 73–146) | Low |
| General population | Chu et al. (2016) | Nepal | Overall incidence: 213 per 1000 person-years  Incidence during RSV epidemic: 443 per 1000 person-years  Incidence rates in infants aged 1-6 months: 107, 246, 222, 256, 220, and 160 per 1000 person-years, respectively  Overall infant hospitalization rate: 2.7 per 1000 person-years | Moderate |
|  | Kamigaki et al. (2017) | Philippines | Incidence rates in outpatients: 1.4 per 1000 persons per year,  RSV hospitalization rate among children aged 6–23-months: 1.9 per 1000 persons per year | Low |
|  | Jayaweera et al. (2021) | Sri Lanka | RSV-ARTI incidence: 0.3 per 1000 persons per year | Moderate |
|  | Mahikul et al. (2019) | Thailand | Incidence of RSV-ALRI hospitalization: 0.31 (0.11–0.34) in 2005 and 0.35 (0.22–0.38) in 2011 per 1000 persons per year | Low |
|  | Chan et al. (2015) | Hong Kong | RSV incidence in children less than 5 years: 15.8 per 1000 persons per year | Low |
|  | Naorat et al. (2013) | Thailand | RSV-associated ALRI: 8.5 per 1000 persons per year | Low |
| Children/Elderly | Naorat et al. (2013) | Thailand | RSV overall hospitalization incidence: 0.85 per 1000 persons per year  In people aged:  <5 years: 9.81 per 1000 persons per year  <1 year: 15.43 per 1000 persons per year  >65 years: 1.3 per 1000 persons per year | Low |
|  | Fry et al. (2010) | Thailand | RSV hospitalization rates in people aged:  <1 year: 10.67 per 1000 persons per year  1-4 years: 4.03 per 1000 persons per year  >65 years: 0.42 per 1000 persons per year | Low |

# Appendix A. Full Search Terms Among 5 Databases.

PubMed Search:

("Respiratory syncytial virus"[Text Word] OR "RSV"[Text Word]) AND (Asia[Text Word] OR India[Text Word] OR China[Text Word] OR Indonesia[Text Word] OR Pakistan[Text Word] OR Bangladesh[Text Word] OR Japan[Text Word] OR Philippines[Text Word] OR Vietnam[Text Word] OR Iran[Text Word] OR Turkey[Text Word] OR Thailand[Text Word] OR Myanmar[Text Word] OR "South Korea"[Text Word] OR Iraq[Text Word] OR Afghanistan[Text Word] OR Yemen[Text Word] OR Uzbekistan[Text Word] OR Malaysia[Text Word] OR "Saudi Arabia"[Text Word] OR Nepal[Text Word] OR "North Korea"[Text Word] OR Syria[Text Word] OR "Sri Lanka"[Text Word] OR Kazakhstan[Text Word] OR Cambodia[Text Word] OR Jordan[Text Word] OR "United Arab Emirates"[Text Word] OR Tajikistan[Text Word] OR Azerbaijan[Text Word] OR Israel[Text Word] OR Laos[Text Word] OR Turkmenistan[Text Word] OR Kyrgyzstan[Text Word] OR Singapore[Text Word] OR Lebanon[Text Word] OR Palestine[Text Word] OR Oman[Text Word] OR Kuwait[Text Word] OR Georgia[Text Word] OR Mongolia[Text Word] OR Qatar[Text Word] OR Armenia[Text Word] OR Bahrain[Text Word] OR Timor-Leste[Text Word] OR Cyprus[Text Word] OR Bhutan[Text Word] OR Maldives[Text Word] OR Brunei[Text Word] OR Taiwan[Text Word] OR "Hong Kong"[Text Word] OR Macao[Text Word]) AND (Seasonality[Text Word] OR Cost*[Text Word] OR Incidence[Text Word])

Web of Science:

((KP=("Respiratory Syncytial Virus" OR RSV)) AND CU=(Asia OR India OR China OR Indonesia OR Pakistan OR Bangladesh OR Japan OR Philippines OR Vietnam OR Iran OR Turkey OR Thailand OR Myanmar OR "South Korea" OR Iraq OR Afghanistan OR Yemen OR Uzbekistan OR Malaysia OR "Saudi Arabia" OR Nepal OR "North Korea" OR Syria OR "Sri Lanka" OR Kazakhstan OR Cambodia OR Jordan OR "United Arab Emirates" OR Tajikistan OR Azerbaijan OR Israel OR Laos OR Turkmenistan OR Kyrgyzstan OR Singapore OR Lebanon OR Palestine OR Oman OR Kuwait OR Georgia OR Mongolia OR Qatar OR Armenia OR Bahrain OR Timor-Leste OR Cyprus OR Bhutan OR Maldives OR Brunei OR Taiwan OR "Hong Kong" OR Macao)) AND ALL=(Seasonality OR Cost* OR Incidence)

Cochrane Library:

"Respiratory Syncytial Virus" OR RSV in All Text AND Asia OR India OR China OR Indonesia OR Pakistan OR Bangladesh OR Japan OR Philippines OR Vietnam OR Iran OR Turkey OR Thailand OR Myanmar OR "South Korea" OR Iraq OR Afghanistan OR Yemen OR Uzbekistan OR Malaysia OR "Saudi Arabia" OR Nepal OR "North Korea" OR Syria OR "Sri Lanka" OR Kazakhstan OR Cambodia OR Jordan OR "United Arab Emirates" OR Tajikistan OR Azerbaijan OR Israel OR Laos OR Turkmenistan OR Kyrgyzstan OR Singapore OR Lebanon OR Palestine OR Oman OR Kuwait OR Georgia OR Mongolia OR Qatar OR Armenia OR Bahrain OR Timor-Leste OR Cyprus OR Bhutan OR Maldives OR Brunei OR Taiwan OR "Hong Kong" OR Macao in All Text AND Cost* OR Seasonality OR Incidence in All Text

Scopus:

(ALL(Respiratory Syncytial Virus OR RSV) AND ALL(Asia OR India OR China OR Indonesia OR Pakistan OR Bangladesh OR Japan OR Philippines OR Vietnam OR Iran OR Turkey OR Thailand OR Myanmar OR South Korea OR Iraq OR Afghanistan OR Yemen OR Uzbekistan OR Malaysia OR Saudi Arabia OR Nepal OR North Korea OR Syria OR Sri Lanka OR Kazakhstan OR Cambodia OR Jordan OR United Arab Emirates OR Tajikistan OR Azerbaijan OR Israel OR Laos OR Turkmenistan OR Kyrgyzstan OR Singapore OR Lebanon OR Palestine OR Oman OR Kuwait OR Georgia OR Mongolia OR Qatar OR Armenia OR Bahrain OR Timor-Leste OR Cyprus OR Bhutan OR Maldives OR Brunei OR Taiwan OR Hong Kong OR Macao) AND ALL(Incidence OR Cost* OR Seasonality))

Embase:

(("respiratory syncytial virus" or RSV) and (Asia or India or China or Indonesia or Pakistan or Bangladesh or Japan or Philippines or Vietnam or Iran or Turkey or Thailand or Myanmar or "South Korea" or Iraq or Afghanistan or Yemen or Uzbekistan or Malaysia or "Saudi Arabia" or Nepal or "North Korea" or Syria or "Sri Lanka" or Kazakhstan or Cambodia or Jordan or "United Arab Emirates" or Tajikistan or Azerbaijan or Israel or Laos or Turkmenistan or Kyrgyzstan or Singapore or Lebanon or Palestine or Oman or Kuwait or Georgia or Mongolia or Qatar or Armenia or Bahrain or "Timor-Leste" or Cyprus or Bhutan or Maldives or Brunei or Taiwan or "Hong Kong" or Macao) and (Seasonality or Cost$ or Incidence)).mp.

# Appendix B. List of Studies Excluded After Full-text Review

| Author (Year) | Title | Reasons for exclusion |
| --- | --- | --- |
| Ching-Hu Chung (2023) | 1746. RSV seasonality patterns change during 2016 to 2020 among hospitalized young children in Taiwan | Poster abstract with no mention of number of RSV cases; and lack of data for seasonality, cost and incidence |
| Dana Danino (2022) | 2203. Involvement of Respiratory Viruses in Community-Acquired Alveolar Pneumonia (CAAP) in Children < 5 Years in Southern Israel, Before and During the COVID-19 Pandemic | Poster abstract with no mention of number of RSV cases; and lack of data for cost and incidence |
| Ron Dagan (2023) | 2618. Four Respiratory Viruses are Involved in the Majority of Community-Acquired Alveolar Pneumonia (CAAP) Episodes in Children &lt; 5 Years | Poster abstract with unsubstantial data |
| Casalegno, Jean-Sebastien (2023) | 889. The age of RSV cases changes consistently over the course of annual epidemics: potential insights into RSV transmission dynamics based on surveillance data from seven countries. | Abstract; insufficient information |
| Kanda Vathanophas (1990) | A Community-Based Study of Acute Respiratory Tract Infection in Thai Children | Paper was too old |
| Ziheng Feng (2023) | A multicentre study on the incidence of respiratory viruses in children with community-acquired pneumonia requiring hospitalization in the setting of the zero-COVID policy in China | No desired outcome (seasonality, incidence, cost) |
| Subharee Suwanjutha (1990) | A Study of Nonbacterial Agents of Acute Lower Respiratory Tract Infection in Thai Children | Paper was too old |
| Malak Elsobky (2024) | Adult respiratory syncytial virus disease burden: systematic literature review in Africa, Asia, Latin America, and the Middle East (2012–2022) | Repetition or redundance of data |
| Tal Brosh-Nissimov (2024) | Adult Respiratory Syncytial Virus Infection: Defining Incidence, Risk Factors for Hospitalization, and Poor Outcomes, a Regional Cohort Study, 2016–2022 | Incomplete or unclear data |
| Fumihiko Ueno (2019) | Age-specific incidence rates and risk factors for respiratory syncytial virus-associated lower respiratory tract illness in cohort children under 5 years old in the Philippines | Incomplete or unclear data |
| Ling Gong (2021) | Analysis of Incidence and Clinical Characteristics of RSV Infection in Hospitalized Children: A Retrospective Study | No desired outcome (seasonality, incidence, cost) |
| Ko et al (2017) | Analysis of Respiratory Viral Infections Detected Using Multiplex Real-Time PCR in Hwaseong, Korea from 2013 to 2015 | No access to full paper |
| Kittikraisak et al (2025) | Antenatal RSV and hMPV illnesses rates among pregnant women in Thailand and association between antenatal RSV and perinatal outcomes: A prospective cohort study | No access to full paper |
| van Houten et al (2018) | Antibiotic Overuse in Children with Respiratory Syncytial Virus Lower Respiratory Tract Infection | No desired outcome (seasonality, incidence, cost) |
| Kabir et al (2009) | ARI situation in our country: aren't we oblivious of bronchiolitis in Bangladesh? | No access to full paper |
| Hosseini et al (2015) | Association between respiratory viruses and exacerbation of COPD: a case-control study | No RSV specific data |
| Huang et al (2022) | Association of children wheezing diseases with meteorological and environmental factors in Suzhou, China | No RSV specific data |
| Pientong et al (2011) | Atypical bacterial pathogen infection in children with acute bronchiolitis in northeast Thailand | No RSV specific data |
| Bhuiyan et al (2017) | Bacterial and viral pathogen spectra of acute respiratory infections in under-5 children in hospital settings in Dhaka city | Too small sample size |
| Nishimura et al (2009) | Breastfeeding reduces the severity of respiratory syncytial virus infection among young infants: a multi-center prospective study | Paper was too old |
| Kayıran et al (2010) | Bronşiyolit tanısıyla izlenen küçük çocuklarda RSV sıklığı, klinik ve laboratuvar özellikleri | Too small sample size |
| Gómez et al (2024) | Burden of Disease Due to Respiratory Syncytial Virus in Adults in Five Middle-Income Countries | No RSV specific data |
| Ohbayashi et al (2024) | Burden of respiratory syncytial virus infections in older adults with acute respiratory infection in Japan: An epidemiological study among outpatients | No desired outcome (seasonality, incidence, cost) |
| Kim et al (2008) | Burden of viral respiratory disease hospitalizations among children in a community of Seoul, Republic of Korea, 1995 - 2005 | No RSV specific data |
| Ji et al (2011) | Characteristics and the prevalence of respiratory viruses and the correlation with climatic factors of hospitalized children in Suzhou children's hospital | No access to full paper |
| Thabet et al (2016) | Characteristics of severe acute respiratory infectionassociated hospitalization in Yemen, 2014/15 | No RSV specific data |
| Omer et al (2008) | Climatic, temporal, and geographic characteristics of respiratory syncytial virus disease in a tropical island population | Paper was too old |
| Huang et al (2017) | Clinical analysis of children with pertussis and significance of respiratory virus detection in the combined diagnosis | No access to full paper |
| Zhang et al (2014) | Clinical characteristics and risk factors of severe respiratory syncytial virus-associated acute lower respiratory tract infections in hospitalized infants | No desired outcome (seasonality, incidence, cost) |
| Zhao et al (2025) | Clinical characteristics of infants with respiratory syncytial virus infection under 3 months of age before, during, and after the SARS-CoV-2 pandemic | No access to full paper |
| Wahab et al (2001) | Clinical characteristics of respiratory syncytial virus infection in hospitalized healthy infants and young children in Qatar | Paper was too old |
| Chu et al (2016) | Clinical Presentation and Birth Outcomes Associated with Respiratory Syncytial Virus Infection in Pregnancy | Too small sample size |
| Agrawal et al (2009) | Comparative evaluation of real-time PCR and conventional RT-PCR during a 2 year surveillance for influenza and respiratory syncytial virus among children with acute respiratory infections in Kolkata, India, reveals a distinct seasonality of infection | No access to full paper |
| Na et al (2025) | Comparison of clinical characteristics and outcomes in hospitalized adult patients infected with respiratory syncytial virus and influenza virus | No desired outcome (seasonality, incidence, cost) |
| Xie et al (2024) | Correction: Seasonality of respiratory syncytial virus infection in children hospitalized with acute lower respiratory tract infections in Hunan, China, 2013-2022 | Repetition or redundance of data |
| Chan and Abdel-Latif et al (2003) | Cost of hospitalization for respiratory syncytial virus chest infection and implications for passive immunization strategies in a developing nation | Paper was too old |
| Dagan et al (2025) | Decline of community-acquired alveolar pneumonia positive for respiratory syncytial virus in hospitalized children following implementation of PCV in Israel | No RSV specific data |
| Li et al (2014) | Detection of respiratory viruses in influenza-like illness in Shijiazhuang, China in 2011 | No access to full paper |
| Wu et al (2014) | Detection of viruses and atypical bacteria associated with acute respiratory infection of children in Hubei, China | No desired outcome (seasonality, incidence, cost) |
| Bozdemir et al (2017) | Direct Medical Cost Assessment in the <2 Years Old Hospitalized RSV+LRTI Patients | No access to full paper |
| Ghia and Rambhad et al (2021) | Disease Burden Due to Respiratory Syncytial Virus in Indian Pediatric Population: A Literature Review | Repetition or redundance of data |
| Zhou et al (2019) | Disease severity and clinical outcomes of community-acquired pneumonia caused by non-influenza respiratory viruses in adults: a multicentre prospective registry study from the CAP-China Network | No RSV specific data |
| Etemadi et al. (2019) | Diversity of respiratory viruses detected among hospitalized children with acute lower respiratory tract infections at Hospital Serdang, Malaysia | Incomplete or unclear data |
| Bhatt and Everard et al (2004) | Do environmental pollutants influence the onset of respiratory syncytial virus epidemics or disease severity? | No RSV specific data |
| Rocha-Filho et al (2023) | Economic burden of respiratory syncytial and parainfluenza viruses in children of upper-middle-income countries: a systematic review | Repetition or redundance of data |
| Guo et al (2023) | Epidemiological and genetic characteristics of respiratory syncytial virus infection in children from Hangzhou after the peak of COVID-19 | No desired outcome (seasonality, incidence, cost) |
| Trifonova et al (2024) | Epidemiological and Genetic Characteristics of Respiratory Viral Coinfections with Different Variants of Severe Acute Respiratory Syndrome Coronavirus 2 (SARS-CoV-2) | Too small sample size |
| Sun et al (2024) | Epidemiological characteristics of human respiratory syncytial virus in influenza-like illness in Shenzhen City from 2019 to 2023 | No access to full paper |
| Su et al (2024) | Epidemiological characteristics of human respiratory syncytial virus in patients with severe acute respiratory infection in Tianjin City from 2015 to 2020 | No access to full paper |
| Wang et al (2013) | Epidemiological characteristics of influenza virus and respiratory syncytial virus among children in Wuhan area from 2008 to 2012 | No access to full paper |
| Ren et al (2021) | Epidemiological characteristics of respiratory syncytial virus in hospitalized children with acute lower respiratory tract infection in Chongqing, China, from 2013 to 2018: an analysis of 2 066 cases | No access to full paper |
| Luo et al (2024) | Epidemiological characteristics of respiratory syncytial virus infection in children in Nanchang and its correlation with climate environmental factors | No access to full paper |
| Chen et al. (2018) | Epidemiology and clinical characteristics of acute respiratory tract infections among hospitalized infants and young children in Chengdu, West China, 2009-2014 | No RSV specific data |
| Chang et al. (2024) | Epidemiology and clinical characteristics of hospitalized adults with respiratory syncytial virus infection at a medical center in northern Taiwan | Too small sample size |
| Al-Toum et al. (2006) | Epidemiology and clinical characteristics of respiratory syncytial virus infections in Jordan | Too small sample size |
| Ramzali et al. (2024) | Epidemiology and clinical features of respiratory syncytial virus (RSV) infection in hospitalized children during the COVID-19 pandemic in Gorgan, Iran | No desired outcome (seasonality, incidence, cost) |
| Kim et al. (2024) | Epidemiology and Disease Burden of Respiratory Syncytial Virus Infection in Adults | No desired outcome (seasonality, incidence, cost) |
| Nguyen et al. (2016) | Epidemiology and etiology of influenza-like-illness in households in Vietnam; it's not all about the kids! | Too small sample size |
| Sung et al. (1993) | Epidemiology and etiology of pneumonia in children in Hong Kong | Paper was too old |
| Bader et al. (2024) | Epidemiology and risk factors associated with bronchiolitis requiring intensive care among children admitted in King Abdulaziz Medical City | Abstract; insufficient information |
| Suryadevara and Domachowske (2021) | Epidemiology and Seasonality of Childhood Respiratory Syncytial Virus Infections in the Tropics | Repetition or redundance of data |
| Ge et al. (2018) | Epidemiology and Seasonality of Respiratory Viruses Detected from Children with Respiratory Tract Infections in Wuxi, East China | No RSV specific data |
| Zhang et al. (2014) | Epidemiology characteristics of respiratory viruses found in children and adults with respiratory tract infections in southern China | No RSV specific data |
| Liang et al. (2023) | Epidemiology dynamic of the common respiratory virus in winter-spring, 2018-2023 in Guangdong province, China | No RSV specific data |
| Lu et al. (2013) | Epidemiology of human respiratory viruses in children with acute respiratory tract infections in Jinan, China | Too small sample size |
| Chan et al. (1999) | Epidemiology of respiratory syncytial virus infection among paediatric patients in Hong Kong: seasonality and disease impact | Paper was too old |
| Hindupur et al. (2019) | Epidemiology of respiratory syncytial virus infections in Chennai, South India | Too small sample size |
| Matsumura et al. (2025) | Epidemiology of respiratory viruses according to age group, 2023-24 winter season, Kyoto, Japan | Too small sample size |
| Khuri-Bulos et al. (2020) | Factors associated with severity of respiratory viral illnesses in hospitalized Jordanian children | Abstract; insufficient information |
| Shan et al. (2024) | Global Seasonal Activities of Respiratory Syncytial Virus Before the Coronavirus Disease 2019 Pandemic: A Systematic Review | Lack of Asia-specific data |
| Sung et al. (2009) | Identification of viral and atypical bacterial pathogens in children hospitalized with acute respiratory infections in Hong Kong by multiplex PCR assays | Too small sample size |
| Kaneko et al. (2002) | Impact of respiratory syncytial virus infection as a cause of lower respiratory tract infection in children younger than 3 years of age in Japan | Paper was too old |
| Deng et al. (2024) | Impact of Subgroup Distribution on Seasonality of Human Respiratory Syncytial Virus: A Global Systematic Analysis | No desired outcome (seasonality, incidence, cost) |
| Subhi et al. (2020) | Impact, epidemiology and clinical manifestations of respiratory syncytial virus in Oman | Abstract; insufficient information |
| Azziz-Baumgartner et al. (2024) | Incidence of laboratory-confirmed influenza and RSV and associated presenteeism and absenteeism among healthcare personnel, Israel, influenza seasons 2016 to 2019 | No desired outcome (seasonality, incidence, cost) |
| Greenberg et al. (2020) | Incidence of respiratory syncytial virus bronchiolitis in hospitalized infants born at 33-36 weeks of gestational age compared with those born at term: a retrospective cohort study | Incomplete or unclear data |
| Langley et al. (2022) | Incidence of Respiratory Syncytial Virus Lower Respiratory Tract Infections During the First 2 Years of Life: A Prospective Study Across Diverse Global Settings | Too small sample size |
| Homaira et al. (2012) | Incidence of respiratory virus-associated pneumonia in urban poor young children of Dhaka, Bangladesh, 2009-2011 | No RSV specific data |
| Weinberger et al. (2013) | Influence of pneumococcal vaccines and respiratory syncytial virus on alveolar pneumonia, Israel | No desired outcome (seasonality, incidence, cost) |
| Miyama et al. (2024) | Irregular seasonality of respiratory syncytial virus infection persists in 2023 in Osaka, Japan | Incomplete or unclear data |
| Bloom-Feshbach et al. (2013) | Latitudinal variations in seasonal activity of influenza and respiratory syncytial virus (RSV): a global comparative review | Paper was too old |
| Li et al. (2021) | National burden estimates of hospitalisations for acute lower respiratory infections due to respiratory syncytial virus in young children in 2019 among 58 countries: a modelling study | No access to full paper |
| Okubo et al. (2025) | Nationwide Epidemiology and Outpatient Healthcare Resource Use of Children with Respiratory Syncytial Virus from 2005 to 2021 | No desired outcome (seasonality, incidence, cost) |
| Nasreen et al. (2014) | Population-based incidence of severe acute respiratory virus infections among children aged <5 years in rural Bangladesh, June-October 2010 | Too small sample size |
| Lan et al. (2024) | Post-Pandemic Epidemiology of Respiratory Infections among Pediatric Inpatients in a Tertiary Hospital in Shanghai, China | Too small sample size |
| Nolan et al. (2015) | Prevalence and Incidence of Respiratory Syncytial Virus and Other Respiratory Viral Infections in Children Aged 6 Months to 10 Years With Influenza-like Illness Enrolled in a Randomized Trial | No RSV specific data |
| Dadashi et al. (2018) | Prevalence of Human Respiratory Syncytial Virus in Iran: A Systematic Review and meta-analysis | No access to full paper |
| Broor et al. (2014) | Rates of respiratory virus-associated hospitalization in children aged <5 years in rural northern India | Too small sample size |
| Sini de Almeida et al. (2024) | Respiratory syncytial virus burden in children under 2 years old in understudied areas worldwide: gap analysis of available evidence, 2012-2022 | No desired outcome (seasonality, incidence, cost) |
| Savic et al. (2023) | Respiratory syncytial virus disease burden in adults aged 60 years and older in high-income countries: A systematic literature review and meta-analysis | No desired outcome (seasonality, incidence, cost) |
| Pangesti et al. (2019) | Respiratory syncytial virus in the Western Pacific Region: a systematic review and meta-analysis | Repetition or redundance of data |
| Albargish and Hasony (1999) | Respiratory syncytial virus infection among young children with acute respiratory tract infection in Iraq | Paper was too old |
| Saijo et al. (1993) | Respiratory syncytial virus infection in lower respiratory tract and asthma attack in hospitalized children in North Hokkaido, Japan | Paper was too old |
| Mosalli et al. (2022) | Respiratory syncytial virus nosocomial outbreak in neonatal intensive care: A review of the incidence, management, and outcomes | Too small sample size |
| Suwanjutha et al. (2002) | Respiratory syncytial virus-associated lower respiratory tract infection in under-5-year-old children in a rural community of central Thailand, a population-based study | Paper was too old |
| Ali et al. (2016) | Respiratory viruses associated with severe pneumonia in children under 2 years old in a rural community in Pakistan | Too small sample size |
| Yeolekar et al (2008) | Respiratory viruses in acute respiratory tract infections in Western India | Paper was too old |
| Hacımustafaoğlu et al. (2013) | RSV frequency in children below 2 years hospitalized for lower respiratory tract infections | No access to full paper |
| Zayed et al. (2024) | SA71 Evaluation of Disease Burden (COMORBIDITIES, LENGTH OF HOSPITALIZATION AND HEALTHCARE RESOURCE UTILIZATION) from Respiratory Syncytial Virus (RSV) Illness in United Arab Emirates: A Retrospective Cohort Claims Database Study | Incomplete or unclear data |
| Chi et al (2018) | Seasonal peaks and risk factors of respiratory syncytial virus infections related hospitalization of preterm infants in Taiwan | Incomplete or unclear data |
| Chan et al. (2002) | Seasonal variation in respiratory syncytial virus chest infection in the tropics | Paper was too old |
| Lee et al (2023) | Seasonality and risk factor analysis of respiratory syncytial virus infection in children in Taiwan-A retrospective study from 1995 to 2005 | Paper was too old |
| Kamigaki et al (2016) | Seasonality of Influenza and Respiratory Syncytial Viruses and the Effect of Climate Factors in Subtropical-Tropical Asia Using Influenza-Like Illness Surveillance Data, 2010 -2012 | Incomplete or unclear data |
| Sayyad et al (2024) | Severe acute respiratory infections: An epidemiological analysis of surveillance data in Bahrain, 2018-2022 | Too small sample size |
| Farfour et al (2021) | Spring is coming, where are the Respiratory Syncytial Virus and Influenza viruses? | Incomplete or unclear data |
| Yassine et al (2020) | Systematic Review of the Respiratory Syncytial Virus (RSV) Prevalence, Genotype Distribution, and Seasonality in Children from the Middle East and North Africa (MENA) Region | Repetition or redundance of data |
| Kurai et al. (2023) | Targeted Literature Review of the Burden of Respiratory Syncytial Infection among High-Risk and Elderly Patients in Asia Pacific Region | Repetition or redundance of data |
| Wan et al. (2023) | The changing pattern of common respiratory viruses among children from 2018 to 2021 in Wuhan, China | No RSV specific data |
| Liu et al. (2021) | The correlation study on human respiratory syncytial virus daily incidence and meteorological parameters in the main urban area of Chongqing from 2009 to 2019 | No desired outcome (seasonality, incidence, cost) |
| Zhang et al. (2023) | The epidemiological features of respiratory tract infection using the multiplex panels detection during COVID-19 pandemic in Shandong province, China | No RSV specific data |
| Chuang et al. (2023) | The Impact of the COVID-19 Pandemic on Respiratory Syncytial Virus Infection: A Narrative Review | Repetition or redundance of data |
| Ekenoglu et al. (2024) | The impact of the COVID-19 pandemic on the circulation, seasonal distribution, and research of other respiratory pathogens in Turkey | No RSV specific data |
| Al Kindi (2023) | Time trend of respiratory viruses before and during the COVID-19 pandemic in severe acute respiratory virus infection in the Sultanate of Oman between 2017 and 2022 | No desired outcome (seasonality, incidence, cost) |
| Yum et al. (2021) | Trends in Viral Respiratory Infections During COVID-19 Pandemic, South Korea | No desired outcome (seasonality, incidence, cost) |
| Ye et al. (2025) | Understanding the local-level variations in seasonality of human respiratory syncytial virus infection: a systematic analysis | No desired outcome (seasonality, incidence, cost) |
| Paynter et al. (2014) | Using mathematical transmission modelling to investigate drivers of respiratory syncytial virus seasonality in children in the Philippines | No desired outcome (seasonality, incidence, cost) |
| Mishra et al. (2016) | Viral Agents Causing Acute Respiratory Infections in Children under Five: A Study from Eastern India | No RSV specific data |
| Chowdhury et al. (2020) | Viral etiology of pneumonia among severely malnourished under-five children in an urban hospital, Bangladesh | Too small sample size |
| Horton et al. (2017) | Viral etiology, seasonality and severity of hospitalized patients with severe acute respiratory infections in the Eastern Mediterranean Region, 2007-2014 | Lack of Asia-specific data |
| Nasreen et al. (2012) | Virus-specific incidence rates of hospitalization for severe acute respiratory infections among children aged <5 years in rural Bangladesh, 2010 | Abstract; insufficient information |
| Meng et al. (2022) | W069 Multicenter study of common pathogen epidemiology in hospitalized children with acute respiratory tract infection in winter from 2017 to 2018, China | Abstract; insufficient information |
| Tariq et al. (2005) | Winter peak of respiratory syncytial virus in Islamabad | No access to full paper |
